# Impact of air pollution on human activities: Evidence from nine million mobile phone users

**DOI:** 10.1371/journal.pone.0251288

**Published:** 2021-05-19

**Authors:** Wei Chen, YingHua He, Shiyuan Pan

**Affiliations:** 1 State Key Lab of CAD&CG, Zhejiang University, Hangzhou, Zhejiang Province, China; 2 Department of Economics, Rice University, Houston, TX, United States of America; 3 CRPE and School of Economics, Zhejiang University, Hangzhou, China; University of Wisconsin Madison, UNITED STATES

## Abstract

To measure the effects of air pollution on human activities, this study applies statistical/econometric modeling to hourly data of 9 million mobile phone users from six cities in China’s Zhejiang Province from December 18 to 21, 2013. Under a change in air quality from “Good” (Air Quality Index, or AQI, between 51 and 100) to “Heavily Polluted” (AQI between 201 to 300), the following effects are demonstrated. (i) Consistent with the literature, for every one million people, 1, 482 fewer individuals are observed at parks, 95% confidence interval or CI (−2, 229, −735), which represents a 15% decrease. (ii) The number of individuals at shopping malls has no statistically significant change. (iii) Home is the most important location under worsening air quality, and for every one million people, 63, 088 more individuals are observed at home, 95% CI (47, 815, 78, 361), which represents a 19% increase. (iv) Individuals are on average 633 meters closer to their home, 95% CI (529, 737); as a benchmark, the median distance from home ranges from 300 to 1900 meters across the cities in our sample. These effects are not due to weather or government regulations. We also provided provisional evidence that individuals engage in inter-temporal activity substitutions within a day, which leads to mitigated (but not nullified) effects of air pollution on daily activities.

## Introduction

Air pollution in China during wintertime consistently draws media attention [1] because of its well-documented adverse health consequences [2–4] and negative impacts on cognitive performance [5]. Those findings are corroborated by evidence from elsewhere. To name a few, [6–9] document a negative effect of air pollution on health/mortality in the U.S.; [10] finds a robust negative relationship between air quality and infant mortality in Africa; [11] shows the impact of exposure to air pollution on student test scores; and [12] demonstrates that outdoor air pollution reduces the productivity of indoor workers.

Therefore, countermeasures by individuals may be naturally implemented against air pollution, such as particulate-filtering facemasks [13] or air purifiers for home use [14]. Our study focuses on how individuals change their activities, including outdoor activities.

To test how outdoor activities change in response to air pollution, the common approach in the literature is to study the attendance at major outdoor facilities, for example, the Griffith Park Observatory and the Los Angeles Zoo & Botanical Gardens in Los Angeles [15, 16], the Bristol Zoo Gardens in Bristol, U.K. [17], and national parks in the U.S. [18]. While these studies convincingly document that individuals avoid these outdoor facilities when air pollution is elevated, they do not inform us what individuals choose to do as a substitute.

Substitutions between different activities can be crucial to measuring the costs of air pollution and thus to developing appropriate policies. For instance, when air pollution is severe, if more individuals choose to go to indoor facilities, such as shopping malls, the negative effects of pollution can be mitigated provided that those facilities supply purified air; however, if individuals choose to be at home, only those who have a well-functioning air purifier can successfully avoid pollution. Different substitution patterns lead to different policy implications.

Our study aims to advance this literature by measuring the substitution patterns in a dataset of nine million mobile phone users in six Chinese cities. In particular, by taking advantage of detailed information on each user’s hourly location, we overcame the main challenge in such a study: observing high-frequency behaviors of a large number of individuals.

We make two contributions to the literature. First and foremost, in terms of the substitution pattern, we demonstrate that individuals, in general, choose to be at home when air pollution is elevated, which has potentially important policy implications. For example, the official recommendations (as detailed later in Table 1) state that when the air quality is “Heavily Polluted,” children, senior citizens, and individuals with respiratory or cardiac issues should *stay indoors*. However, such a recommendation may not be sufficient because indoor air quality can also be poor. Not every home is well insulated against outdoor pollutants [19], and indoor air quality can be made worse by cigarette smoking and cooking at home [20–22] or chemicals from furniture and decorations [23]. Several studies, as reviewed by [24], show that the inflow of outdoor pollutants, combined with internal sources (e.g. indoor combustion, particle resuspension) can make air quality lower than outdoors. Moreover, high-income households are more likely to purchase air purifiers for their homes [14]; thus, air pollution may further exacerbate inequalities in health status. In contrast, shopping malls, museums, schools, and other public indoor facilities in China have started to take measures to clean their indoor air [25, 26]. Helping individuals monitor indoor air quality, especially at home, and encouraging individuals to stay at an indoor facility with clean air may more effectively reduce the adverse effects of air pollution on health.

**Table 1:** AQI categorization and MEP recommendations.

AQI	Air Quality	Health Implications	Official Recommendations
0–50	Excellent	Air quality is satisfactory with almost no air pollution.	Daily activities are free of air pollution concerns.
51–100	Good	Air quality is acceptable but certain pollutants can have mild health impacts on vulnerable individuals.	Hypersensitive individuals should reduce outdoor activities.
101–150	Slightly Polluted	Symptoms of vulnerable individuals may elevate, and slight irritations may occur among healthy people.	Children, senior citizens, and individuals with respiratory or cardiac issues should reduce intense, long-duration outdoor exercises.
151–200	Moderately Polluted	Symptoms of vulnerable individuals can be further worsened, and there may be impacts on respiratory or cardiac systems of healthy people.	Children, senior citizens, and individuals with respiratory or cardiac issues should avoid intense, long-duration outdoor exercises. Everyone else should reduce outdoor exercises.
201–300	Heavily Polluted	Individuals with respiratory or cardiac issues will experience noticeably worse symptoms and reduced endurance. Healthy people will also have health issues.	Children, senior citizens, and individuals with respiratory or cardiac issues should stay indoors. Everyone else should further reduce outdoor exercises.
> 300	Severely Polluted	Healthy people will experience reduced endurance and have noticeable and severe symptoms. Certain illnesses will be triggered.	Children, senior citizens, and individuals with health issues should stay indoors and avoid physical activities. Everyone else should avoid outdoor activities.

*Notes*: Authors’ translation from the official document in Chinese published by MEP (Source: https://web.archive.org/web/20131227173225/http://kjs.mep.gov.cn/hjbhbz/bzwb/dqhjbh/jcgfffbz/201203/W020120410332725219541.pdf, archived on December 27, 2013). Each level of air quality is color-coded by MEP, corresponding to the colors in the table.

Second, our modeling of human behavior in a big dataset might be useful to others conducting related research. Our massive dataset of mobile phone logs contains rich information on phone users’ locations; however, it is also contaminated with significant noise. Modeling individual location choice and performing appropriate aggregation enable the flexible control of noise and measurement errors while reducing the computational burden. As the availability of big datasets increases rapidly, our strategy provides some insights when a tractable way of dealing with such datasets is required.

### Geolocating mobile phones

More specifically, our data cover six cities in Zhejiang Province of China, namely, Huzhou, Jiaxing, Ningbo, Taizhou, Wenzhou, and Zhoushan, from December 18 to 21, 2013. To investigate the effects of air pollution, we focused on each phone user’s location in each hour and his/her distance from home.

We geolocated each mobile phone in a given hour by using the locations of the mobile phone towers, or Base Transceiver Stations, to which it connected, which is similar to [27]. However, this method differs from that in studies that rely on mobile-phone locating-request data from certain mobile apps, such as those from Tencent [28], Baidu [29], and Facebook [30], or location-based service data (e.g., geo-tagged messages) from social media, such as Twitter [31] or Weibo [4]. During the COVID-19 pandemic, mobility data from commercial vendors are used to investigate the impacts of the pandemic, e.g., [32]; such data are also different from ours because a vendor may use a mobile phone’s GPS, the name of its connected WiFi network, and even the MAC address of its connected router to geolocate a phone. More details about one of the vendors, Unacast, are available at https://www.unacast.com/privacy.

An advantage of our dataset is that it covers a less selected sample of individuals in the six cities. In our sample period, the mobile phone service penetration rates in these cities range from 125% to 212% [33], and our dataset contains *all* the users in these cities from the biggest service provider, which had a national market share of 62.42% in 2013, calculated based on the total number of subscribers announced in the provider’s 2013 annual financial report and the national total number of subscribers in [34]. Our dataset is far from being a representative sample of all adults in those cities because these phone users may be different from non-users and users with other providers. We discuss the potential issues that this non-representativeness can bring in the sensitivity analyses. However, our dataset does not suffer from certain common selection issues. For example, a mobile app generates a location data point only if a phone user has installed the app, allows it to use location services, and launches the app; therefore, only a self-selected subsample of mobile phone users can be observed in datasets based on app location data.

A disadvantage of our dataset is the potential errors in geolocating a phone. A location is often serviced by multiple towers, and a phone at that location can connect to any of them. In the geolocating procedure, we assume that within an hour, a phone connects to a tower for a duration proportional to the distance between them (relative to the phone’s distances to other towers). There are many reasons that this assumption can be violated. For example, a tower may reach its capacity and refuse to allow more connections. Our modeling approach guarantees that our estimation is consistent as long as the measurement errors are not correlated with air pollution. The sensitivity analyses discuss possible biases when the errors and air pollution are correlated.

### Addressing other potential issues

We aim to measure how individuals voluntarily respond to air pollution rather than factors like weather and government regulations. As clarified in the “Materials and methods” section, during our sample period, major weather events were not observed and the six cities did not have regulations on human mobility related to air pollution. An additional important factor is individuals’ knowledge about real-time air pollution levels. In regular weather reports on TV and radio, the official air quality level for the city and occasionally an exact air quality reading for the past hour was reported. More detailed information was available online. Because the city-level air quality measure was arguably the most salient to individuals, we used it as the main explanatory variable.

Similar to any study that examines the effects of area-based attributes on individual behaviors, our study faces the uncertain geographic context problem (UGCoP) [35], which helps identify two sources of contextual uncertainty in our setting: uncertainty in the spatial configuration of the appropriate units for assessing the effects of air pollution and uncertainty about the timing and duration of exposure to the unit’s air pollution. The spatial unit in our study is a city in the administrative sense. As city-level air pollution measured by the air quality index is the most commonly publicized, individuals are more likely to respond to this information. Further, our study assumes that individuals react to air pollution on an hourly basis. These assumptions rule out the possibility that individuals only care about air pollution in their neighborhood or adjust their reactions to air pollution more/less frequently than hourly.

In summary, we conducted a range of sensitivity analyses and provided some caveats in interpreting our results; in particular, we addressed the following concerns: (i) a phone’s location and distance from home are measured with error; (ii) a single mobile phone user may hold multiple phones; (iii) our data may not be representative; and (iv) including different explanatory variables, e.g., alternative measures of air quality, may affect our results. Our results are shown to be reasonably robust.

### Summary of findings

We categorized the locations into four mutually exclusive groups, namely, home, park, shopping mall, and others, and modeled individual location choice as a logit discrete choice problem. The model was applied to our data, and we found that increased air pollution is associated with more individuals being at home. Under a scenario in which air quality worsens from “Good” (the second-best level according to the official standard) to “Heavily Polluted” (the second-worst level), we demonstrated the following effects of air pollution: (i) For every one million people, 1, 482 fewer of them are observed at parks, 95% CI (−2, 229, −735), which represents a 15% decrease; (ii) the number of individuals at shopping malls does not show a significant response to such a change in air pollution; (iii) home is the most important location when there is such a worsening in air pollution, and for every one million people, 63, 088 more individuals are observed at home, 95% CI (47, 815, 78, 361), which amounts to a 19% increase; and (iv) in the time window from 7:00 to 22:00, the above effects are less pronounced from 13:00 to 17:00.

We also estimated the effects of air pollution on individuals’ distance from home with a Tobit model [36]. The results demonstrate that when the air quality worsens from “Good” to “Heavily Polluted,” individuals are 633 meters closer to their home on average, 95% CI (529, 737), which represents a sizable reduction because the median distance from home ranges from 400 to 1900 meters across different cities from 7:00 to 22:00 (or 300 to 1900 meters during all hours).

Additionally, we analyzed daily data and found provisional evidence consistent with the following: (i) individuals engage in intra-day substitutions of activities and (ii) the intra-day substitution mitigates but does not nullify the effects of air pollution on human activities. Therefore, if air pollution only peaks for a couple of hours within a day, the results indicate that the foregone benefits of some behaviors, such as leaving home, can be partially recouped because people can choose to leave home in the hours when air pollution is low.

### Outline of the article

The next section describes the data. We then describe how we constructed hourly data on individual activities and modeled location choice and distance from home. After we present the empirical findings, the penultimate section discusses potential issues and sensitivity analyses. Finally, the last section concludes.

## Materials and methods

### Data and background information

We now provide a description of the data and some background information, such as how air pollution information was announced to the public. We argue that weather and government regulations are unlikely to be the driving forces of our findings.

We relied on two types of data: (i) hourly air quality data and (ii) mobile phone tower logs. We focused on four days from December 18 to 21, 2013, and six cities in Zhejiang Province of China: Huzhou, Jiaxing, Ningbo, Taizhou, Wenzhou, and Zhoushan. Public holidays or special celebrations did not occur in any of the cities during the sample period.

**Fig 1:**
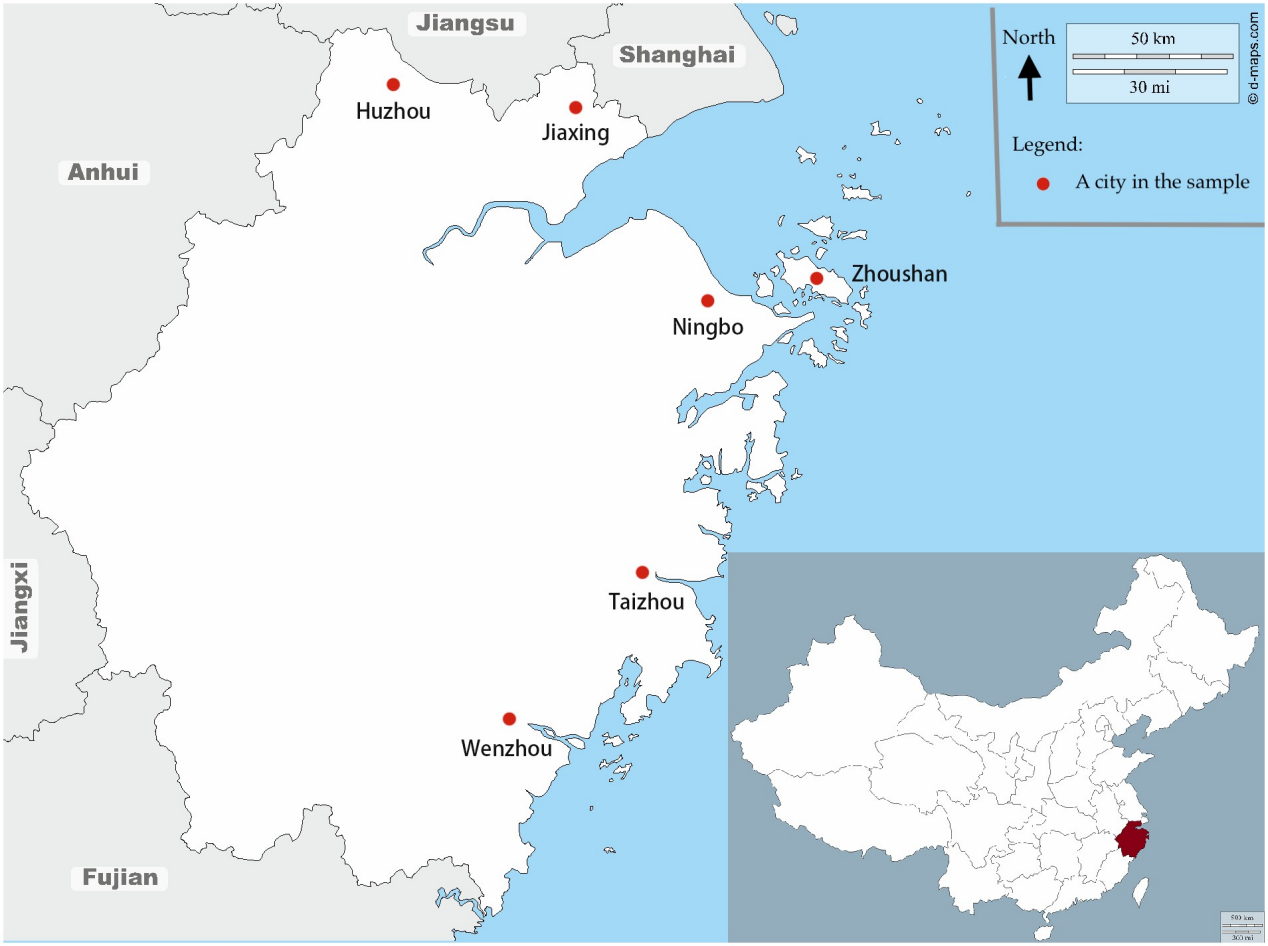
Six cities in Zhejiang Province, China. This map is produced by the authors based on two maps downloaded from https://d-maps.com/carte.php?num_car=20248 and https://d-maps.com/carte.php?num_car=17500. The copyright holder of the original maps has granted use of this map in this article (https://d-maps.com/conditions.php).

As Fig 1 shows, these cities are spread out across the province, and they also vary in size and economic development. All of them are relatively wealthy in China, with a per capita GDP in 2013 from 49, 817 yuan (Wenzhou) to 123, 139 yuan (Ningbo), or 8, 229 to 20, 340 US dollars. In comparison, the GPD per capita at the national level was 43, 684 yuan in 2013. All cities have high levels of mobile phone service penetration rate, ranging from 1.25 (Taizhou) to 2.12 (Ningbo) subscriptions per resident [33]. For more details, see S1 Table in S1 File.

#### Data on air quality

We collected hourly air pollution data for the six cities. The main air pollution measure is the hourly AQI. In China, the Ministry of Environmental Protection (MEP) monitors the level of outdoor air pollution in major cities. The AQI is calculated based on the levels of six atmospheric pollutants recorded at the monitoring stations within each city, including sulfur dioxide (SO_2_), nitrogen dioxide (NO_2_), suspended particulates smaller than 10 *μm* in aerodynamic diameter (PM_10_), suspended particulates smaller than 2.5 *μm* in aerodynamic diameter (PM_2.5_), carbon monoxide (CO), and ozone (O_3_). The AQI provides the maximum values among the indices that are based on these pollutants. The exact formula and calculation method are available on the MEP website (in Chinese): https://web.archive.org/web/20131227173225/http://kjs.mep.gov.cn/hjbhbz/bzwb/dqhjbh/jcgfffbz/201203/W020120410332725219541.pdf, (archived on December 27, 2013). These standards were published in 2012 and adopted by Zhejiang Province before our sample period. In addition, we also collected hourly data on PM_2.5_ and PM_10_, which are the hourly average levels of PM_2.5_ and PM_10_ (both in *μg*/*m*^3^).

A given AQI reading leads to an official pollution-level category, and the MEP publishes its associated health implications and activity recommendations (Table 1). Weather forecasts usually report the hourly AQI and the associated activity recommendations in their regular reports, which may prompt changes in individual activities. For instance, when the AQI is between 51 and 100 (i.e., the air quality is “Good”), it is advised that “[h]ypersensitive individuals should reduce outdoor activities.” When air quality worsens to “Heavily Polluted” (AQI between 201 and 300), it is recommended that “[c]hildren, senior citizens, and individuals with respiratory or cardiac issues should stay indoors. Everyone else should further reduce outdoor exercises.”

**Fig 2:**
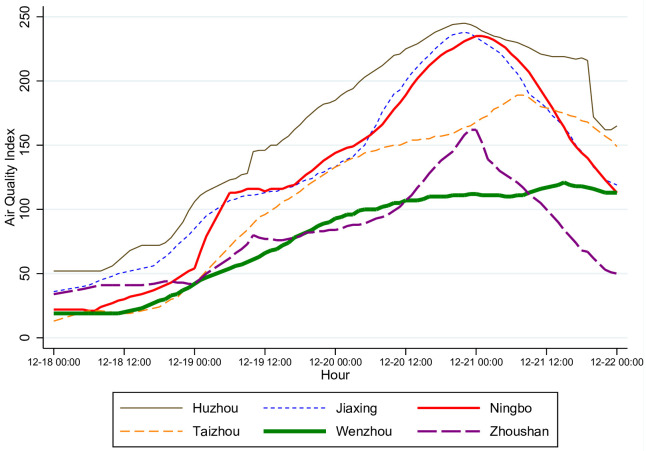
Hourly Air Quality Index (AQI) in the six cities from December 18–21, 2013. This figure plots the official, city-level AQI for each city. Data source: (https://www.aqistudy.cn/).

Our sample period, December 18 to 21, 2013, included a wave of air pollution. Fig 2 shows that the AQI ranged from 25 to 225 and peaked on December 20 to 21. The pollution followed the usual pattern, spreading from the north to the south, with inland areas suffering more than coastal cities. The AQIs in Huzhou and Jiaxing in northern Zhejiang peaked the earliest, and those in Taizhou and Wenzhou in southern Zhejiang peaked the latest. Overall, Huzhou, Jiaxing, and Ningbo experienced worse air pollution than the other three cities.

#### Mobile phone tower records

We obtained anonymized mobile phone logs from the biggest mobile phone service provider in China. Each data point is a mobile tower record. Whenever a phone connects to or disconnects from, a tower (or Base Transceiver Station), it generates a record with a time stamp. If a phone user uses a mobile service (e.g., cellular data, phone call, or text messaging) while being connected to a tower, the system also creates a new record. Each record indicates the reason for its creation.

Our sample includes all records from all mobile phone towers located in the six cities for the period 20:00 December 17 (Tuesday) to 06:00 December 22 (Sunday). Necessarily, the raw data include many temporary visitors in the cities; therefore, we imposed some sample selection rules. The analysis only included mobile phone users that (i) have at least four active days (i.e., the duration between its first and last records in the period from December 17 to 22 is at least 96 hours) and (ii) have at least 20 mobile tower records. These restrictions were imposed on the raw data. The excluded phone users are highly likely to be visitors or residents who went out of town during our sample period. In addition, we excluded the two partial days in the data, and our main sample covers four full days: 00:00 December 18 (Wednesday) to 24:00 December 21 (Saturday).

Similar to the air quality data, we transformed the mobile phone records into hourly data. On average, 1.5 million phone numbers were observed in an hour in each city. Table 2 provides more details on the summary statistics.

**Table 2:** Number of hourly mobile phone users: Summary statistics.

City	Mean	Standard deviation	Min	Max
Huzhou	624,708	2,940	606,185	625,613
Jiaxing	1,114,119	3,313	1,093,764	1,115,134
Ningbo	2,509,575	4,004	2,482,290	2,510,725
Taizhou	1,622,502	3,302	1,601,377	1,623,502
Wenzhou	3,048,195	3,655	3,022,082	3,049,246
Zhoushan	137,642	338	135,659	137,748
Overall	1,509,457	1,017,556	135,659	3,049,246

*Notes*: The data are hourly during the four days from December 18–21, 2013. For each city, there are 96 observations.

#### Information, weather, and regulations

*Information on air pollution*. In regular weather reports on TV and radio, the official level of air quality and occasionally the exact AQI reading for the past hour are reported. The associated activity advice, as documented in Table 1, was also announced. When the AQI is above 50, or the air quality is worse than “Excellent,” the top pollutant is reported. In our sample, the main pollutants were PM_2.5_ and PM_10_ (see their hourly averages in S1 Fig in S1 File). PM_2.5_ has been documented to pose various negative health effects, while PM_10_ is considered less harmful; for a summary, see Table ES-1 in [37].

Each city’s hourly AQI takes into account the reports from its monitoring stations. Shortly after the hour, each station submits a detailed hourly report including 9 measures: the averages of *SO*_2_, *NO*_2_, *O*_3_, *CO*, PM_10_, and PM_2.5_ during the past hour, as well as the 8-hour moving average of *O*_3_ and the 24-hour moving averages of PM_10_ and PM_2.5_ [38]. A city maps its stations’ hourly reports into a single hourly AQI. Note that the 24-hour moving averages of PM_10_ and PM_2.5_ are used in the calculation of an hourly AQI [38]. The public can access hourly reported PM_10_ and PM_2.5_ measures through certain websites; however, hourly air quality levels and hourly AQIs, both city-level, are far more accessible to the public and more broadly reported. For these reasons, we use hourly AQIs, or their corresponding air quality levels, as our main measure of air pollution.

Air quality forecast for the entire day is also common because the movement of air pollution in Zhejiang Province is sometimes predictable [39]. In usual forecasts, an expected air pollution level for the next 24 hours is usually reported.

*Weather*. Weather conditions can affect human behaviors as well; however, during our sample period, there were no major weather conditions in any of the six cities. We collected weather data containing information on weather conditions, e.g., precipitation and wind speed. Such information was reported every three hours for each city. Rain or showers were only recorded for 2.7% of the observations. The lowest temperature across all days and all cities, which was usually observed during the hours 0:00 to 6:00, was −3°C; and the highest, which was often observed during the hours 12:00 to 17:00, was 11°C. The average of the daily low temperatures was 0.3°C, and the average of the daily high temperatures was 8.3°C. Besides, 52% of the observations recorded wind with a speed no more than 4 meters per second, which is no stronger than Beaufort scale 3 or a “gentle breeze,” while 89% had winds with a speed no more than 9 meters per second, which is no stronger than Beaufort scale 5 or a “fresh breeze.” The summary statistics are in S4 Table in S1 File. In the sensitivity analyses, we control for the weather conditions in the regressions.

*Regulations on human mobility*. A city may have regulations that directly restrict human behavior when air pollution is severe. However, in 2013, there were no regulations on transportation or vehicle usage on days with high air pollution in Zhejiang Province and discounts were not provided for the use of public transportation on such days. Zhejiang implemented its first set of such regulations on March 1, 2014, as documented on the government’s official website: https://web.archive.org/web/20140406185613/http://www.zj.gov.cn/art/2013/12/27/art_12451_129061.html (in Chinese, archived on April 6, 2014). To our knowledge, there were no regulations on street cleaning or school/work arrangements during high air pollution days in 2013. Because of the lack of such regulations, we are more confident that our findings are due to people’s voluntary reactions to air pollution.

### Measuring individual activities

We used the mobile phone data to geolocate each phone user. Because the information in the data is limited due to privacy protection, we focus on two types of human activities in each one-hour window: (i) a user’s location choice among four mutually exclusive options; namely, the user’s home, a park, a shopping mall, and other locations, and (ii) the distance between the user’s current location and his/her home. The geolocating tasks were carried out in 2014 and 2015.

#### Geolocating mobile phones

Identifying each phone user’s home address is crucial for our analysis; however, our data do not directly contain this information. For a given phone user, we used the following procedure to determine the home address:

(i) Find each mobile phone tower’s geographic coordinates in latitude and longitude degrees (up to five digits after the decimal point, i.e., in Zhejiang Province, the distance precision is up to one meter).
(ii) Identify the towers to which the phone ever connected during the hours 0:00 to 6:00 on any day in our raw data sample period.
(iii) Calculate a user’s home address as the weighted average of the locations of its registered towers, expressed as geographic coordinates in latitude and longitude degrees. The weight for a given tower is the fraction of time that the phone connected to it.

Due to the high density of towers, the majority of mobile phones were connected to multiple towers within the time frame specified above, thus making step (iii) necessary. The underlying assumption is that the connection time between a tower and a phone is proportional to the distance between them relative to the phone’s distances to other ever-registered towers. The same approach is used in [40], who provide more details on this procedure.

Using the information on the towers to which a phone had been connected in a given hour, we followed the same procedure to locate the phone within that hour. After having identified a phone’s home address and current location in each hour, we then calculated the distance between its current location and the user’s home.

**Fig 3:**
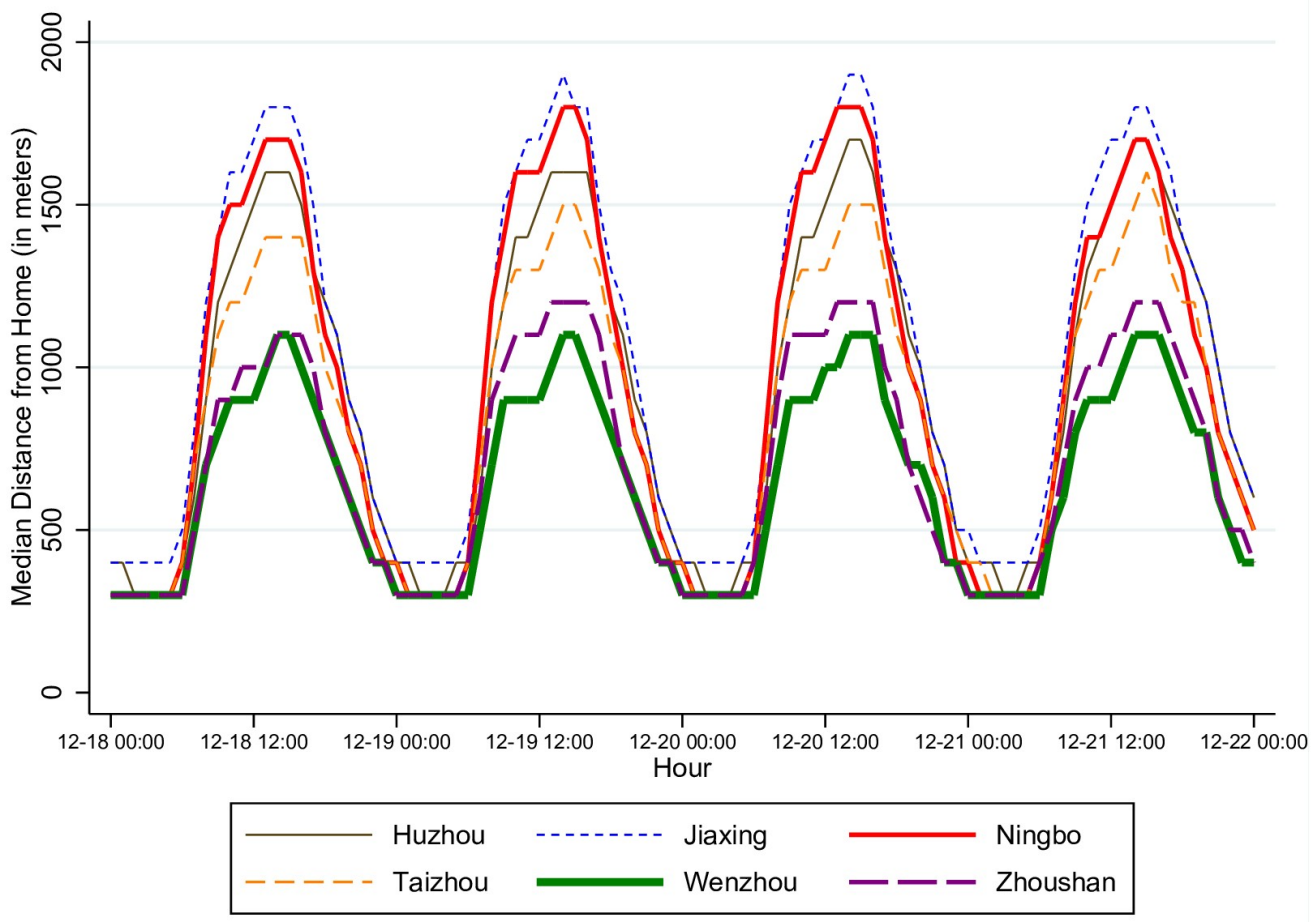
Median distance from home by hour in each city from December 18 to 21, 2013.

Fig 3 shows the median distance from home in each hour. As anticipated, the distance displays a strong time trend within each day, with the lowest level from 23:00 to 7:00 and the peaks from 12:00 to 15:00. Significant spatial variations are observed across cities.

Note that the lowest median distance from home within a day is approximately 300 meters, which may seem surprising because one might expect more than half the population to be at home from 1:00 to 5:00. As explained in the procedure for geolocating, certain measurement errors may have occurred in the identification of home address and current location. In the following analysis, we take these errors into account explicitly. For example, we define that an individual is “at home” if the distance from the home is less than 500 meters. Our results are not sensitive to changes in this definition. Nonetheless, this criterion for determining “at home” is selected to balance the fraction of individuals at home in the late night and early morning hours, e.g., 1:00 to 5:00, and the fraction of individuals at home in the afternoon hours, e.g., 13:00 to 17:00. With 500 meters as the threshold, the former fraction is on average 0.63 (min 0.48; max 0.71), while the latter fraction is on average 0.26 (min 0.19; max 0.35). In comparison, from 1:00 to 5:00, the median distance from home is from 300 to 400 meters across all cities (Fig 3).

Relating the patterns in air quality and distance (Figs 2 and 3), we do not see a clear correlation. For example, the median distance on December 20, when air quality was at its worst level, is not noticeably different from that on other days. This lack of correlation may be due to confounding factors or measurement errors, and our modeling will allow us to control for these factors.

#### Parks and shopping malls

As the guidelines in Table 1 indicate, outdoor activities are discouraged when air quality is poor. We partially measured outdoor activities by the presence of phone users in parks. In addition, we also used visits to shopping malls as an indoor activity and investigated whether air pollution has heterogeneous effects on these two types of activities. One may be tempted to identify each phone user’s workplace and examine work-related activities. Unfortunately, the data do not allow us to identify this information. One of the reasons is that a user’s workplace, e.g., an office building, is usually small relative to a park or a shopping mall.

We located parks and shopping malls in each city with maps from map.baidu.com. As parks are usually color-coded in green, we applied the tool *Grabcut* to identify each park’s boundary [41] and then checked the results manually. With the map of mobile phone towers, we used Voronoi diagrams to identify each tower’s service area, which is its Voronoi cell. Voronoi diagrams are well known for this purpose; for a survey, see [42]. We then overlapped the map of the towers with the map of the parks. If a tower’s service area has at least a five-percent overlap with any park, we consider it a “park tower.” The choice of this seemingly low threshold of five percent is based on the following reasons: (i) A tower’s service area is usually much larger than a small park, (ii) a big park is usually served by several towers, and (iii) an individual may have to travel through a nearby area to get to a park. For a mobile phone away from home, if it connects to one or multiple “park towers” more than 40 minutes within the hour, we define the phone as “at a park” in that hour. It should be emphasized that to identify a phone at a park, we used all its records *before* transforming them into hourly data.

To geolocate shopping malls, we used the following procedure. We performed searches on map.baidu.com, and the keywords were in Chinese, e.g., “shopping” (*gòu wù*), “shopping mall” (*mài cháng*), or well-known shopping malls in Zhejiang Province (e.g., *yín tài*). After locating them on the map, we used the same criteria for parks to determine whether a tower is a “shopping mall tower” and whether a phone user is at a shopping mall. Again, we used a phone’s records *before* transforming them into hourly data.

The numbers of identified parks and shopping malls in each city are summarized in S2 Table in S1 File. These numbers might be lower bounds of the actual ones because we only included those that were identified clearly on the Baidu map.

**Fig 4:**
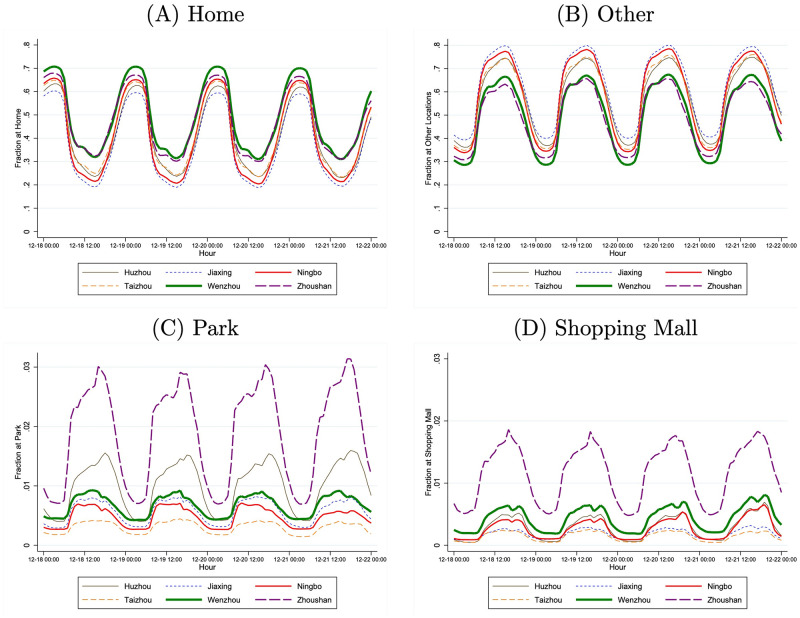
Location of mobile phone users: Hourly shares from December 18–21, 2013.

Based on the above calculations, Fig 4 presents the hourly shares of phone users in each of the four types of locations: home, park, shopping mall, and others. Recall that an individual is defined to be “at home” if he or she is not more than 500 meters away from the estimated home location. A phone user can only be at one location during any given hour. Again, each of the four time series shows a strong time trend within each day. However, the effect of air quality on individual activities does not present a visually noticeable pattern.

Fig 4 also shows the small fractions of people visiting parks or shopping malls. Across all six cities and during any hour of our sample period, the fraction of individuals observed at parks peaks at approximately 3% (Panel C) while the fraction of those being observed at a shopping mall is always less than 2% (Panel D). Therefore, focusing on parks or malls will only provide information on a small share of the population, which implies the importance of considering the other two types of locations, home and other locations.

#### A first look at the data: Saturday vs. weekdays

Our main sample includes four days, Wednesday–Saturday, December 18–21, 2013. One may be concerned that human activities are significantly different between Saturday and other weekdays and that it might bias our estimation given the time trend of air pollution in Fig 2. For this reason, as detailed below, our statistical/econometric model explicitly accounts for the differences between different days, so that the estimated effects of air pollution will not be driven by the differences between Saturday and other days.

On the other hand, the commonly observed differences in human activities between Saturday and weekdays also give us a way to check if our geolocation procedure produced something reasonable. For each hour from 7:00 to 22:00 on each day, we compared individuals’ location choice on Saturday to their average location choice on Wednesday to Friday. The detailed results are in S2 Fig in S1 File, and we summarize the main results as follows. (i) Relative to the same hour on other days, there were significantly more people staying home on Saturday morning, while significantly fewer stayed home from 16:00 to 22:00. This pattern is consistent with the observation that on Saturday, some people may sleep in and stay home in the morning and go out later in the day. (ii) The hourly differences between Saturday and other days in the fraction of people in parks increase over time, from being negative in the morning to being positive in the late afternoon and evening. (iii) Relative to the same hour on other days, the fraction of people at shopping malls is higher only after 13:00 on Saturday. In summary, these findings do not signal any obvious problem with our geolocation process.

### Statistical/Econometric model for human activities

The above descriptive analysis of individual activities motivates us to consider a more sophisticated approach that will allow us to control confounding factors, such as time trends and measurement errors. Below, we present a statistical/econometric model for location choice, followed by another model for distance from home in each hour.

#### Location choice

Formally, we consider a set of individuals, *i* = 1, …, *n*_*j*,*y*,*t*_, who reside in city *j* (*j* ∈ {1, …, 6}) on day *y* (*y* = 1, …, 4) at hour *t* (*t* = 7, 8, …, 22). Here, *t* indicates the hour between *t* − 1 and *t*. We excluded hours between 23:00 and 6:00 of the following day because most people rest at home during these hours. Each *i* faces the choice of activities on day *y* at every time window of one hour, *t*.

We adopt a discrete choice model to investigate individuals’ choice of location. Furthermore, due to the large size of our dataset and potential measurement errors, we will transform the data on individual choices into shares of individuals choosing different options. This transformation brings several advantages, which will be elaborated shortly.

Individual *i* in city *j* chooses a type of location on day *y* at time *t* such that

$$\begin{array}{c} z_{i,j,y,t,l}=1\;\text{if}\;\text{and}\;\text{only}\;\text{if}\;l\in L\;\text{is}\;\text{chosen,} \end{array}$$ (1)

where the choice set of location types is L={h,m,p,o}, with *h* = home, *m* = shopping mall, *p* = park, and *o* indicating others, or the residual locations (that are away from home but not an identified park or shopping mall). Recall that an individual will be at exactly one of the locations in any given hour.

Suppose that individual *i* maximizes his or her utility in each hour and *i*’s utility if choosing *l* is denoted as *w*_*i*,*j*,*y*, *t*,*l*_:

$$\begin{array}{cc} w_{i,j,y,t,l} & =\gamma_{j,y,l}^{\left(L\right)}+\delta_{t,l}^{\left(L\right)}+X_{j,y,t}\beta_{l}^{\left(L\right)}+\eta_{j,y,t,l}+\upsilon_{i,j,y,t,l},\;\text{if}\;l\neq h,\\ w_{i,j,y,t,l} & =\upsilon_{i,j,y,t,l},\;\text{if}\;l=h; \end{array}$$

where $\gamma_{j,y,l}^{\left(L\right)}$ is a city-day-location-specific fixed effect and can be considered the average popularity of location *l* in city *j* on day *y*; $\delta_{t,l}^{\left(L\right)}$ is an hour-location-specific fixed effect and measures the average popularity of location *l* at time *t*; *η*_*j*,*y*,*t*,*l*_ is a city-day-hour-location-specific fixed effect and captures other unobserved factors affecting the popularity of location *l* in city *j* on day *y* at time *t*, including potential measurement errors and the average preference for location *l* among that city’s residents; *X*_*j*,*y*,*t*_ is a row vector of characteristics of city *j* on day *y* at time *t*, including air pollution measures (i.e., dummy variables for different air quality levels or AQI as a continuous variable); and *υ*_*i*,*j*,*y*,*t*,*l*_ is the unobserved heterogeneity at the individual level.

Note that the various fixed effects are designed to account for the potential particularities of Saturday and certain measurement errors in the geolocation procedure. More precisely, in addition to *η*_*j*,*y*,*t*,*l*_, the city-day-location-specific fixed effects ($\gamma_{j,y,l}^{\left(L\right)}$) absorb the differences between a weekend and weekdays as well as measurement errors that are systematically associated with a city in a given day for a location type (e.g., parks or shopping malls). Further, $\delta_{t,l}^{\left(L\right)}$, being hour-location-specific, captures measurement errors that may be possibly larger in certain hours for certain location types.

Moreover, {*υ*_*i*,*j*,*y*,*t*,*l*_}_*l*∈*L*_ are i.i.d. type-I extreme values (Gumbel distribution); thus, the choice probability can be characterized as follows: if *l* = *h*,

$$\begin{array}{cc} & E[z_{i,j,y,t,l}|X_{j,y,t},{\left\{\gamma_{j,y,l^{\prime }}^{\left(L\right)},\delta_{t,l^{\prime }}^{\left(L\right)},\eta_{j,y,t,l^{\prime }}\right\}}_{l^{\prime }\in L}]\\ = & P[z_{i,j,y,t,l}=1|X_{j,y,t},{\left\{\gamma_{j,y,l^{\prime }}^{\left(L\right)},\delta_{t,l^{\prime }}^{\left(L\right)},\eta_{j,y,t,l^{\prime }}\right\}}_{l^{\prime }\in L}]\\ = & \frac{1}{1+\sum_{l^{\prime }\in \left\{m,p,o\right\}}\exp\left(\gamma_{j,y,l^{\prime }}^{\left(L\right)}+\delta_{t,l^{\prime }}^{\left(L\right)}+X_{j,y,t}\beta_{l^{\prime }}^{\left(L\right)}+\eta_{j,y,t,l^{\prime }}\right)}; \end{array}$$

if *l* ∈ {*m*, *p*, *o*},

$$\begin{array}{cc} & E[z_{i,j,y,t,l}|X_{j,y,t},{\left\{\gamma_{j,y,l^{\prime }}^{\left(L\right)},\delta_{t,l^{\prime }}^{\left(L\right)},\eta_{j,y,t,l^{\prime }}\right\}}_{l^{\prime }\in L}]\\ = & P[z_{i,j,y,t,l}=1|X_{j,y,t},{\left\{\gamma_{j,y,l^{\prime }}^{\left(L\right)},\delta_{t,l^{\prime }}^{\left(L\right)},\eta_{j,y,t,l^{\prime }}\right\}}_{l^{\prime }\in L}]\\ = & \frac{\exp(\gamma_{j,y,l}^{\left(L\right)}+\delta_{t,l}^{\left(L\right)}+X_{j,y,t}\beta_{l}^{\left(L\right)}+\eta_{i,j,y,l})}{1+\sum_{l^{\prime }\in \left\{m,p,o\right\}}\exp\left(\gamma_{j,y,l^{\prime }}^{\left(L\right)}+\delta_{t,l^{\prime }}^{\left(L\right)}+X_{j,y,t}\beta_{l^{\prime }}^{\left(L\right)}+\eta_{j,y,t,l^{\prime }}\right)}. \end{array}$$

Let *n*_*j*,*y*,*t*,*l*_ be the *observed* total number of individuals in city *j* choosing location *l* on day *y* at time *t*. When the total population, *n*_*j*,*y*,*t*_, is large, then the law of large numbers implies:

$$\begin{array}{cc} \frac{n_{j,y,t,h}}{n_{j,y,t}}= & \frac{1}{1+\sum_{l^{\prime }\in \left\{m,p,o\right\}}\exp\left(\gamma_{j,y,l^{\prime }}^{\left(L\right)}+\delta_{t,l^{\prime }}^{\left(L\right)}+X_{j,y,t}\beta_{l^{\prime }}^{\left(L\right)}+\eta_{j,y,t,l^{\prime }}\right)};\\ \frac{n_{j,y,t,l}}{n_{j,y,t}}= & \frac{\exp(\gamma_{j,y,l}^{\left(L\right)}+\delta_{t,l}^{\left(L\right)}+X_{j,y,t}\beta_{l}^{\left(L\right)}+\eta_{i,j,y,l})}{1+\sum_{l^{\prime }\in \left\{m,p,o\right\}}\exp\left(\gamma_{j,y,l^{\prime }}^{\left(L\right)}+\delta_{t,l^{\prime }}^{\left(L\right)}+X_{j,y,t}\beta_{l^{\prime }}^{\left(L\right)}+\eta_{j,y,t,l^{\prime }}\right)},\;\text{for}\;l\in \left\{m,p,o\right\}. \end{array}$$

These equations further imply the following equation for the log odds ratio,

$$\begin{array}{cc} \ln\left(n_{j,y,t,l}\right)-\ln\left(n_{j,y,t,h}\right) & =\ln\left(n_{j,y,t,l}/n_{j,y,t}\right)-\ln\left(n_{j,y,t,h}/n_{j,y,t}\right)\\ & =\gamma_{j,y,l}^{\left(L\right)}+\delta_{t,l}^{\left(L\right)}+X_{j,y,t}\beta_{l}^{\left(L\right)}+\eta_{j,y,t,l},\;\text{for}\;l\in \left\{m,p,o\right\}. \end{array}$$ (2)

Eq (2) thus formulates a linear regression for the location choice data.

Transforming the individual-level discrete choice model in Eq (1) into the aggregated linear regression in Eq (2) brings several advantages. First, it reduces the computation burden dramatically. At the individual level, there are more than 500 million total data points across the six cities and four days, and we would need to estimate at least 1152 *η*_*j*,*y*,*t*,*l*_’s as well as other coefficients. In contrast, the aggregated regression, Eq (2), allows for separate estimations for each location and only requires us to work with 384 hourly observations (6 cities, 4 days, and 16 hours per day). Second, by including $\gamma_{j,y,l}^{\left(L\right)}$, $\delta_{t,l}^{\left(L\right)}$, and *η*_*j*,*y*,*t*,*l*_, the aggregated model conveniently allows for measurement errors when calculating the number of individuals at each type of location, which is useful in our mobile phone data because each can represent systematic or random noise in the geolocating of mobile phones (in city *j*, day *y*, hour *t*, and location type *l*).

In our main specification, we include four dummy variables for different levels of air pollution (*Excellent*_*j*,*y*,*t*_, *Slightly*_*Polluted*_*j*,*y*,*t*_, *Moderately*_*Polluted*_*j*,*y*,*t*_, and *Heavily*_*Polluted*_*j*,*y*,*t*_), each of which equals one if the air quality in city *j* at hour *t* of day *y* is at that level and zero otherwise. Although there are six possible levels of air quality in total as shown in Table 1, our data do not include any hour in which air quality was rated as “Severely Polluted” (or AQI above 300, see Fig 2 and S3 Table in S1 File). Moreover, the air quality of “Good” (AQI between 51 and 100) is the omitted benchmark level and a coefficient for an included air quality dummy variable, therefore, measures the difference between that level and “Good.”

When we interpret the effects of air pollution, marginal effects are more helpful. We focus on the effects of air quality changes from “Good” to “Heavily Polluted” (AQI between 201 and 300), while other comparisons can be computed in the same manner. One reason to focus on these two levels is that the former represents a usual good air day while the latter represents when air pollution becomes more salient amongst the public. For *l* ∈ {*m*, *p*, *o*}, the effect on the share of individuals choosing location *l* is measured as follows:

$$\begin{array}{cc} & \frac{1}{384}\sum_{j,y,t}{\frac{\exp(\gamma_{j,y,l}^{\left(L\right)}+\delta_{t,l}^{\left(L\right)}+X_{j,y,t}\beta_{l}^{\left(L\right)}+\eta_{i,j,y,l})}{1+\sum_{l^{\prime }\in \left\{m,p,o\right\}}\exp\left(\gamma_{j,y,l^{\prime }}^{\left(L\right)}+\delta_{t,l^{\prime }}^{\left(L\right)}+X_{j,y,t}\beta_{l^{\prime }}^{\left(L\right)}+\eta_{j,y,t,l^{\prime }}\right)}|}_{Heavily\_Polluted_{j,y,t}=1}\\ - & \frac{1}{384}\sum_{j,y,t}{\frac{\exp(\gamma_{j,y,l}^{\left(L\right)}+\delta_{t,l}^{\left(L\right)}+X_{j,y,t}\beta_{l}^{\left(L\right)}+\eta_{i,j,y,l})}{1+\sum_{l^{\prime }\in \left\{m,p,o\right\}}\exp\left(\gamma_{j,y,l^{\prime }}^{\left(L\right)}+\delta_{t,l^{\prime }}^{\left(L\right)}+X_{j,y,t}\beta_{l^{\prime }}^{\left(L\right)}+\eta_{j,y,t,l^{\prime }}\right)}|}_{Good_{j,y,t}=1}, \end{array}$$ (3)

where ${\cdot |}_{Heavily\_Polluted_{j,y,t}=1}$ is the expression being evaluated at *Heavily*_*Polluted*_*j*,*y*,*t*_ = 1 (meaning that all other air quality dummies are equal to zero) and ${\cdot |}_{Good_{j,y,t}=1}$ is the expression being evaluated at *Good*_*j*,*y*,*t*_ = 1 (meaning that all other air quality dummies are equal to zero). In other words, for each hourly observation, we calculate *Heavily*_*Polluted*_*j*,*y*,*t*_ = 1 and *Good*_*j*,*y*,*t*_ = 1 while taking other variables at their observed value. The marginal effect is the average difference between the two values across all hourly observations. The effect of the same change in air pollution on the share of individuals being at home is similarly calculated.

As a robustness check, instead of dummy variables for air quality levels, we will include the AQI as a continuous measure in *X*_*j*,*y*,*t*_ (Eq 2). When the AQI increases, the associated marginal effects on the choice probabilities of different locations are as follows:

$$\begin{array}{cc} \frac{\partial (n_{j,y,t,l}/n_{j,y,t})}{\partial AQI_{j,y,t}}= & \frac{n_{j,y,t,l}}{n_{j,y,t}}\beta_{l,AQI}^{\left(L\right)}-\frac{n_{j,y,t,l}}{n_{j,y,t}}(\sum_{l^{\prime }\in \left\{m,p,o\right\}}\beta_{l^{\prime },AQI}^{\left(L\right)}\frac{n_{j,y,t,l^{\prime }}}{n_{j,y,t}}), \end{array}$$ (4)

$$\begin{array}{cc} \frac{\partial (n_{j,y,t,h}/n_{j,y,t})}{\partial AQI_{j,y,t}}= & -\frac{n_{j,y,t,h}}{n_{j,y,t}}(\sum_{l^{\prime }\in \left\{m,p,o\right\}}\beta_{l^{\prime },AQI}^{\left(L\right)}\frac{n_{j,y,t,l^{\prime }}}{n_{j,y,t}}), \end{array}$$ (5)

where $\beta_{l,AQI}^{\left(L\right)}$ is the coefficient of *AQI*_*j*,*y*,*t*_ in the utility function of location *l*.

#### Distance from home

Complementary to the location choice analysis, we also studied an individual’s distance from home during each hour. Suppose that we find more individuals being observed at home instead of at parks when air pollution worsens. If we also document that individuals are on average much closer to their home at the same time, then the reduction in the distance from home provides a proxy to their valuation of being at parks. In other words, without air pollution, they would have been willing to travel the distance to go to a park. This distance analysis also provides an indirect test on the sensitivity of the location choice results, e.g., relative to the definition of being at home.

Individual *i*’s intended distance from home is determined by the following equation:

$$\begin{array}{c} d_{i,j,y,t}^{*}=\gamma_{j,y}^{\left(D\right)}+\delta_{t}^{\left(D\right)}+X_{j,y,t}\beta^{\left(D\right)}+\xi_{j,y,t}+\varepsilon_{i,j,y,t}, \end{array}$$ (6)

where $d_{i,j,y,t}^{*}\in R$ is the intended distance from home that *i* in city *j* intends to have on day *y* at time *t*; $\gamma_{j,y}^{\left(D\right)}$ is the average intended distance in city *j* on day *y* (e.g., city *j* may have more people preferring out-of-home activities on average on day *y* than another day and/or other cities); $\delta_{t}^{\left(D\right)}$ measures the intended distance at time *t* (e.g., individuals are more likely to be away from home during certain hours); *X*_*j*,*y*,*t*_ is a row vector of characteristics of city *j* on day *y* at time *t*, similar to that in the location choice model; *ξ*_*j*,*y*,*t*_ includes unobserved factors in city *j* on day *y* at time *t*; and lastly, *ε*_*i*,*j*,*y*,*t*_ follows a normal distribution and captures unobserved factors and measurement errors at the individual level. Moreover, each of $\gamma_{j,y}^{\left(D\right)}$, $\delta_{t}^{\left(D\right)}$, and *ξ*_*j*,*y*,*t*_ can capture certain systematic errors in measuring the distance from home.

Similar to the location choice model, the various fixed effects ($\gamma_{j,y}^{\left(D\right)}$, $\delta_{t}^{\left(D\right)}$, *ξ*_*j*,*y*,*t*_) in the distance model are designed to account for the potential particularities of Saturday and certain measurement errors in the geolocation procedure.

To measure distance from home, we construct 501 intervals, [0, 100], (100, 200], …, (49900, 50000], (50000, ∞), measured in meters. For each individual, we find the interval to which he or she belongs. His or her distance from home is then defined as the mid-point of the interval if the distance is not more than 50 kilometers. Note that the observed distance variable is censored at 50 kilometers. That is, everyone who is at least 50 kilometers away from home is grouped together.

The impossibility of negative distance leads to the observed distance from home (in meters) as follows:

$$\begin{array}{c} d_{i,j,y,t}=\{\begin{array}{cc} 0 & \text{if}\;d_{i,j,y,t}^{*}\leq 0;\\ d_{i,j,y,t}^{*} & \text{if}\;0<d_{i,j,y,t}^{*}\leq 50,000;\\ 50,000 & \text{if}\;d_{i,j,y,t}^{*}>50,000. \end{array} \end{array}$$ (7)

Eqs (6) and (7) naturally lead to a Tobit model that explicitly deals with the censoring at the lower and upper limits [36].

## Results

We first present the results from the location choice analysis based on dummy variables for air quality levels and then those based on the AQI as a continuous measure. The results from the distance analysis are also grouped into two sets with different air quality measures. When discussing marginal effects, we maintain the assumption that every mobile phone user has one phone and that our data on mobile phone users provide a representative sample of the six cities’ population. We discuss the consequences of relaxing these assumptions in the next section.

### Location choice

From the estimation of Eq (2) with the sample of hourly data from 7:00 to 22:00 for the four days, we consider a benchmark estimate in which *X*_*j*,*y*,*t*_ only includes the air quality dummies. The estimation results for different locations (home, park, shopping mall, and others) are in S6 Table in S1 File.

Applying these results to Eq (3), we obtain the marginal effects of air quality worsening from “Good” to “Heavily Polluted” in Panel A of Table 3. This finding shows that such an increase in air pollution is mainly associated with more individuals being at home. In an average city on an hourly basis, the increased air pollution leads to a 6.31 percentage point increase in the share of individuals being at home, 95% CI (4.78, 7.84), amounting to a 19.09% increase. Averaging over the six cities, such an increase in air pollution is associated with 63, 088 more individuals at home per one million mobile phone users, 95% CI (47, 815, 78, 361).

**Table 3:** Effects of air pollution on location choice.

Location	Changes in percentage points		Percent Change		Changes in #individuals per 1 million	
	Point estimate	95% CI	Point estimate	95% CI	Point estimate	95% CI
*Panel A. Air quality changes from “Good” to “Heavily Polluted”*						
Home	6.31	(4.78,7.84)	19.09	(14.47, 23.71)	63,088	(47,815, 78,361)
Other	-6.09	(-7.56, -4.63)	-9.31	(-11.55, -7.07)	-60,909	(-75,563, -46,255)
Park	-0.15	(-0.22, -0.07)	-14.69	(-22.09, -7.28)	-1,482	(-2,229, -735)
Shopping Mall	-0.07	(-0.16, 0.02)	-13.28	(-30.04, 3.48)	-697	(-1,577, 182)
*Panel B. A 10-Point Increase in the AQI (the AQI as a continuous measure)*						
Home	0.68	(0.56, 0.80)	2.05	(1.69, 2.41)	6,770	(5,585, 7,955)
Other	-0.65	(-1.10, -0.21)	-1.00	(-1.68, -0.31)	-6,536	(-11,019, -2,054)
Park	-0.02	(-0.03, -0.01)	-1.63	(-2.64, -0.62)	-164	(-266, -63)
Shopping Mall	-0.01	(-0.02, 0.00)	-1.33	(-2.92, 0.27)	-70	(-153, 14)

*Notes*: The marginal effects are calculated under the assumption that our data on mobile phone users include a representative sample from the population of the six cities. Panel A reports the marginal effects of air quality changing from “Good” to “Heavily Polluted” on individuals’ location choice, and it is calculated by evaluating Eq (3) based on the estimates of Eq (2) (summarized in column 1 of S5 Table in S1 File). The reported confidence intervals are calculated from 500 bootstrap samples. Panel B reports the marginal effects of a 10-point increase in the AQI on individuals’ location choice, and it is calculated by evaluating Eqs (4) and (5) at the sample means and the estimates of Eq (2) (summarized in column 1 of S6 Table in S1 File).

As an often-studied outdoor activity, visits to a park decrease when air quality worsens from “Good” to “Heavily Polluted.” The share of individuals observed at parks, on average, decreases by 0.15 percentage points, 95% CI (0.07, 0.22), a 14.69% decrease. However, because fewer individuals among the population are visiting a park (Fig 4), the reduction in the number of individuals at parks is mere 1, 482 per one million.

We also see a reduction in the share of individuals visiting a shopping mall, although the estimates are not significantly different from zero at the 5% significance level. The individuals at a shopping mall only account for a small fraction of the population (Fig 4). If anything, the effect of air quality changing from “Good” to “Heavily Polluted” on visits to a mall is small in absolute terms, with an average reduction of 697 individuals per one million.

Not surprisingly, air quality worsening from “Good” to “Heavily Polluted” has a large effect on the residual group of away-from-home activities. As Panel A of Table 3 shows, this worsening in air quality leads to a reduction of 60, 909 individuals who would have been away from home but not at a park or a shopping mall, 95% CI (46, 255, 75, 563), per one million people.

Taken together, the above results imply that only a partial picture would have been obtained had we solely focused on the popularity of specific outdoor or indoor activities when studying the effects of air pollution. The most important location for individuals responding to air pollution is their home.

**Fig 5:**
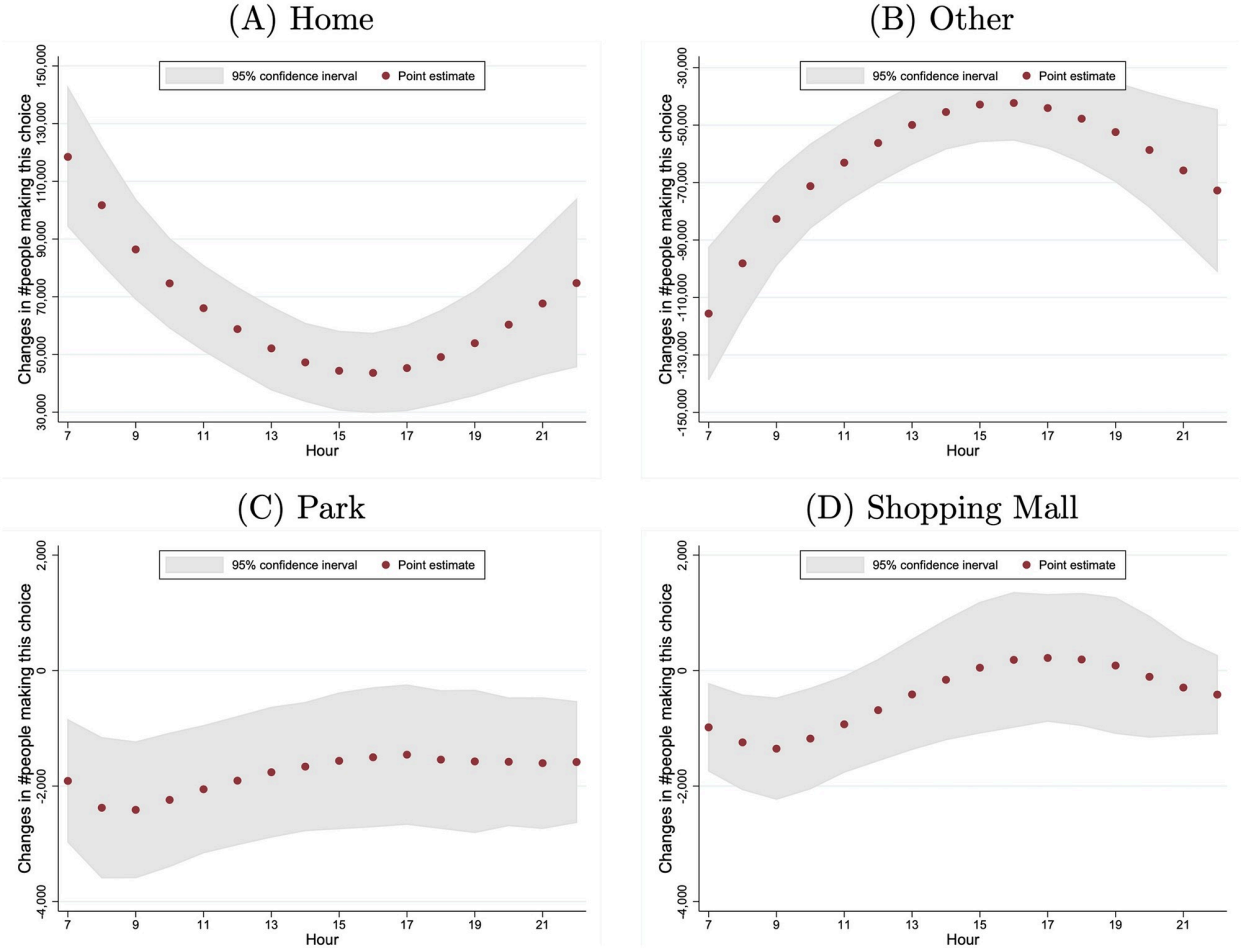
Marginal effects on location choice: Air quality worsening from “good” to “heavily polluted”. This figure presents the hourly marginal effects of air quality worsening from “Good” to “Heavily Polluted” on individuals’ location choice, and it is calculated by evaluating Eq (3) using the estimates of Eq (2) (summarized in column 2 of S5 Table in S1 File). The number of individuals in absolute terms is calculated among one million people under the assumption that the mobile phone users are a random sample from the population. The 95% confidence intervals are constructed from 500 bootstrap samples.

The effects of air pollution on location choice may vary across hours of the day. Therefore, we let *X*_*j*,*y*,*t*_ include the air-quality dummy variables as well as their interactions with *t* and *t*^2^ to allow for the time-varying effects of air pollution. The regression results are in column (2) of S5 Table in S1 File and were used to generate Fig 5. The hourly marginal effects of air quality worsening from “Good” to “Heavily Polluted” are measured by the average number of individuals per one million who are induced to change behavior. The effects on being at home or being at other locations mirror each other: they are higher in the early hours and late evening. The effects on being at parks or shopping malls do not exhibit a strong time trend.

#### Measuring air quality continuously by the AQI

As a way to check the robustness of our results, we estimated Eq (2) with the AQI as a continuous measure, which is useful as some individuals may pay attention to the exact reading of the AQI in addition to the official level of air quality. The results are in column (1) of S6 Table in S1 File. We obtained the marginal effects of a 10-point increase in the AQI and collected the results in Panel B of Table 3.

The results are similar to those in Panel A of the same table. A 10-point increase in the AQI is mainly associated with an increase in the number of individuals being at home. In an average city on an hourly basis, an additional 10 AQI points lead to 0.68 percentage points increase in the share of individuals being at home, amounting to a 2.05% increase, or 6, 770 more individuals per one million. At the same time, the share of individuals being at parks on average decreases by 0.02 percentage points, a 1.63% decrease or a reduction of 164 individuals per one million. Similar to the results with air quality dummy variables, the reduction in individuals being at shopping malls is not significantly different from zero at the 5% significance level. Lastly, a 10-point increase in the AQI is associated with a reduction of 6, 536 individuals per one million who would have been away from home, but not at a park or a shopping mall.

**Fig 6:**
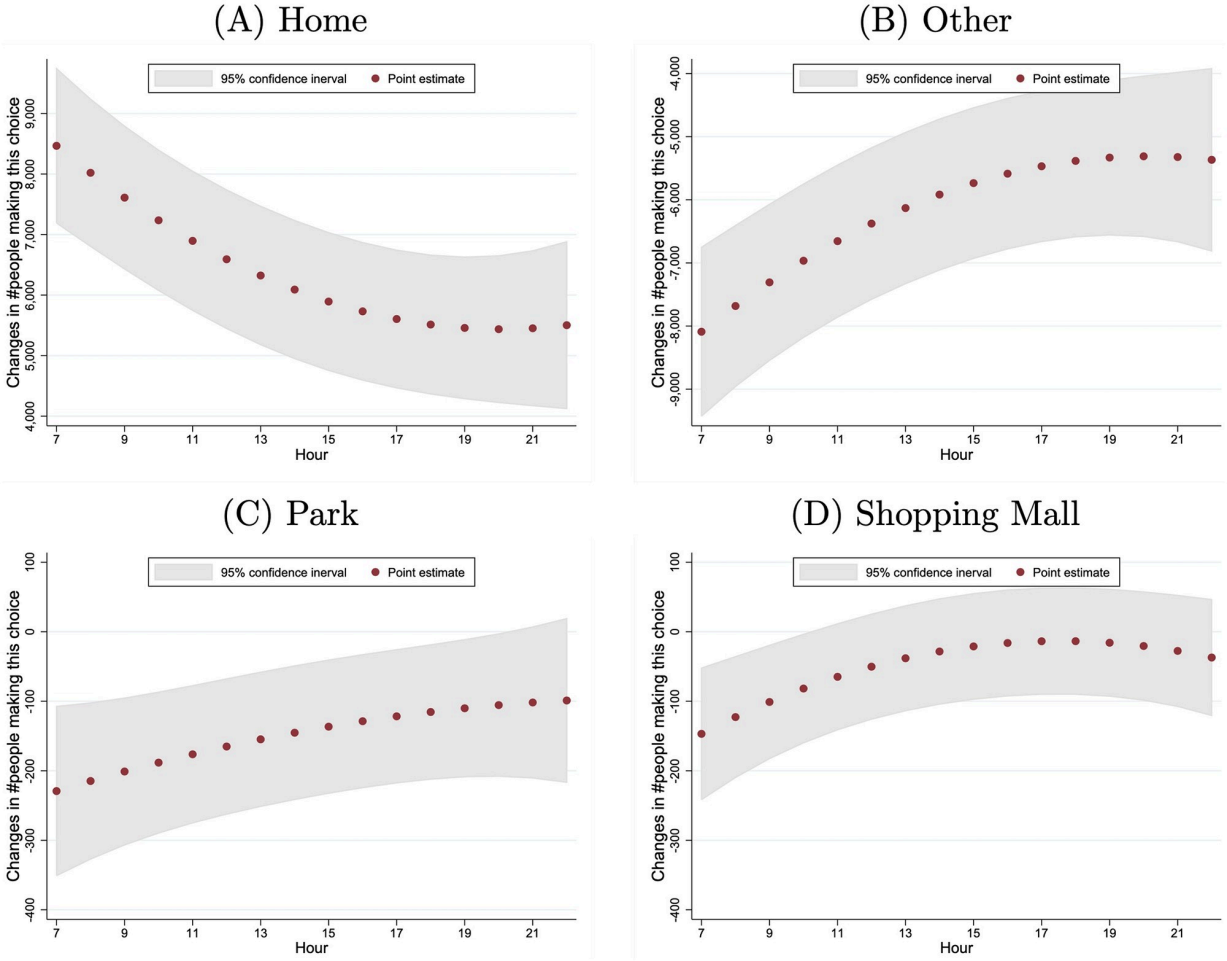
Marginal effects of a 10-point increase in the AQI on location choice. This figure presents the marginal effects of a 10-point increase in the AQI on individuals’ location choice hour by hour, and it is calculated by evaluating Eqs (4) and (5) at the sample means and the estimates of Eq (2) (which are summarized in column 2 of S6 Table in S1 File). The number of individuals in absolute terms is calculated for every one million individuals.

Similarly, we also investigated the heterogeneity in the effect of air pollution across the 16 hours of the day. Fig 6 shows the hourly marginal effects of a 10-point increase in the AQI, measured by the average number of people who are induced to change behavior. The patterns are very similar to those in Fig 5, which are derived from the estimation with air quality dummies.

### Distance from home

We now turn to how air pollution affects an individual’s distance from home. In summary, we found evidence consistent with the results of the location choice analysis: when air pollution is worsened, the average distance from home decreases.

From the estimation of Eq (6) using the sample of hourly data from 7:00 to 22:00 for the four days, we first let *X*_*j*,*y*,*t*_ only include the air quality dummies (*Excellent*_*j*,*y*,*t*_, *Slightly*_*Polluted*_*j*,*y*,*t*_, *Moderately*_*Polluted*_*j*,*y*,*t*_, and *Heavily*_*Polluted*_*j*,*y*,*t*_). The results are in column (1) of S7 Table in S1 File. These findings show that when air quality worsens from “Good” to “Heavily Polluted,” an individual’s distance from home is reduced by 633 meters on average, 95% CI (529, 737), which is a sizable reduction relative to the hourly median distance from home (Fig 3). Across the six cities, the median distance from home, which peaks in the early afternoon, is between 300 and 1, 900 meters, implying that giving up on going to a park or other locations may involve a non-negligible reduction of an individual’s welfare.

**Fig 7:**
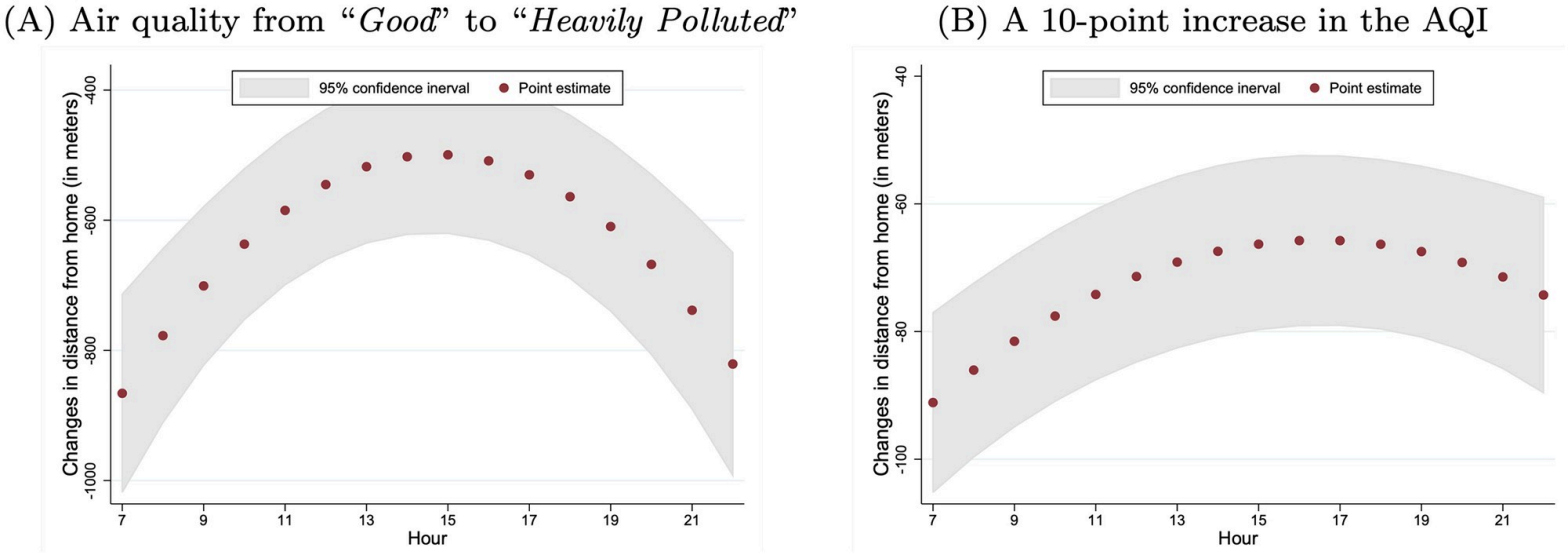
Hourly marginal effects of air pollution on distance from home. Panel A presents the marginal effects of air quality changing from “Good” to “Heavily Polluted” on individuals’ distance from home hour by hour. It is calculated with the estimates of Eq (6), which are summarized in column (2) of S7 Table in S1 File. Panel B presents the marginal effects of a 10-point increase in the AQI on individuals’ distance from home hour by hour, and it is calculated with the estimates of Eq (6), which are summarized in column (2) of S8 Table in S1 File.

Similar to the location analysis, we also explored the time-varying effects of air pollution. We let *X*_*j*,*y*,*t*_ include the air-quality dummy variables, as well as their interactions with *t* and *t*^2^. The regression results are in column (2) of S7 Table in S1 File and lead to Panel A of Fig 7. The hourly marginal effects of air quality worsening from “Good” to “Heavily Polluted,” which is measured by the average changes in distance from home, range from 499 to 866 and are higher in the early morning and late evening and reach the lowest values around noon, which is also consistent with the results from the location choice analysis.

We repeated the analysis with the AQI as a continuous measure. The estimates are reported in column (1) of S8 Table in S1 File. When the AQI increases by 10 points, an individual’s distance to home is reduced on average by 79 meters, 95% CI (66, 91). When we let *X*_*j*,*y*,*t*_ in Eq (7) be *AQI*_*j*,*y*,*t*_, *t* × *AQI*_*j*,*y*,*t*_, and *t*^2^ × *AQI*_*j*,*y*,*t*_, the hourly marginal effects of a 10-point increase in the AQI are depicted in Panel B of Fig 7. They range from 66 to 91 meters and have a pattern similar to those in Panel A.

### Inter-temporal substitution within a day

An individual may plan his or her daily activities at the beginning of the day. Moreover, the pattern of air pollution is usually predictable, especially for waves similar to the one in our data. An air pollution wave usually originates from the North China Plain and spreads out to various parts of China, including Zhejiang Province, which is located in the south [39]. An individual may choose to simply delay or advance rather than canceling out-of-home activities due to air pollution. We considered this intra-day temporal substitution from two angles.

First, the hourly effects of air pollution reaching the lowest level during the afternoon hours implies that the intra-day substitution alone cannot explain our findings. As shown in Fig 2, for most of the days in our sample, the six cities experienced increasing air pollution within a day. If many individuals planned their activities at the beginning of the day to minimize the exposure to air pollution, we would expect that the effects of air pollution would have been larger in later hours when air pollution was generally worse. However, Figs 5–7 show that the estimated effects of air pollution are in general U-shaped within a day, reaching the minimum from 13:00 to 17:00.

Second, if the inter-temporal substitution completely explains an individual’s hourly activities, then we would then observe that individuals adjust their activities in response to hourly changes in air pollution while the daily average would remain constant. To test this hypothesis, we carried out analyses of daily data.

Due to anonymization for privacy protection, our dataset for statistical analyses does not allow us to link users over time, although we could link them in the geolocating procedure. Therefore, we considered alternative methods of testing the hypothesis. In short, we found provisional evidence for an imperfect intra-day substitution of activities.

S9 Table in S1 File presents information on the location choice at the daily level. The dependent variable is the average hourly share of individuals at location *l* (*l* being home, park, shopping mall, or others) within each day. Specifically, for city *j* on day *y*, we calculated $\frac{1}{16}\sum_{t=7}^{22}\frac{n_{j,y,t,l}}{n_{j,y,t}}$ (which is multiplied by 1000 in the regressions). This variable will not be affected by hourly air pollution if an individuals’ inter-temporal substitution of different activities within a day is perfect. We regressed this variable for each location on several measures of daily air quality while controlling for day and city fixed effects. We experimented with a variety of daily AQI measures: daily maximum (column 1), daily mean (column 2), daily median (column 3), and official daily value (column 4). We did not use the air quality dummies because the data do not provide us with many variations in air pollution levels. Due to the small sample size, we assume that the error term in the regression is i.i.d. normal. The table reports significance for two-sided tests.

The results in S9 Table in S1 File indicate provisional evidence for imperfect intra-day substitutions. An increase in air pollution, regardless of the measure we use, is associated with an increase in the share of individuals at home and a reduction in the share of individuals at parks, shopping malls, or others. While almost all the coefficients are not significant (possibly due to the small sample size), the magnitude of each coefficient is generally smaller than the marginal effects from the hourly data, which are reported in Panel B of Table 3. For example, when there is a 10-point increase in the hourly AQI, Table 3 shows a 0.68-percentage-point increase in the share of individuals at home; and when the AQI increases by 10 points in each hour of the day, S9 Table in S1 File shows that the share of individuals at home increases by 0.04 to 0.09 percentage points, which is a much smaller effect.

Furthermore, S10 Table in S1 File studies the daily distance from home at the city level. The dependent variable is the average hourly distance from home each day. Specifically, for city *j* on day *y*, we calculate $\frac{1}{16}\sum_{t=7}^{22}\frac{1}{n_{j,y,t}}\sum_{i=1}^{n_{j}}d_{i,j,y,t}$. The coefficients of air quality measures are always negative, which is consistent with the results from the analysis of hourly data. When there is a 10-point increase in the AQI in every hour of a day, the table shows a decrease in distance from home ranging from 18 to 34 meters, which is also smaller than what we obtained in the analysis of hourly data (i.e., 79 meters).

In summary, the analysis of daily data shows provisional evidence consistent with the following: (i) individuals engage in intra-day substitutions of activities and (ii) the intra-day substitution mitigates but does not nullify the effects of air pollution on human activities. Moreover, due to data limitations, our analysis of daily data is far from conclusive; thus, additional research is needed.

## Potential issues and sensitivity analyses

Our estimation and results present several potential concerns. For example, our constructed measures on location and distance from home contain error; a single user may own multiple mobile phones; the sample of our mobile phone users is not necessarily representative of the population of the six cities or the population of China; our results may be sensitive to alternative regression specifications. In the following, we discuss these concerns.

### Measurement errors in location and distance data

Naturally, our constructed measures on location and distance from home contain errors. We discuss how various errors may bias our results. Let us start with the analysis of location choice.

First, the measurement errors appear in the dependent variables of Eq (2) and thus are contained in *η*_*j*,*y*,*t*,*l*_, which implies that our estimation of the effects of air pollution remains consistent if, conditional on other controls, the measurement errors and the air quality measures are *independent*. In this case, as the measurement errors add noise to our data, the standard errors are inflated but the point estimates remain consistent.

Second, in addition to the aforementioned independent measurement errors, our location measures also have systematic errors. For example, the people in an office building co-located with a shopping mall will be counted as being at a mall when they are actually at work. The part of these errors that are not affected by air quality can be controlled by $\gamma_{j,y,l}^{\left(L\right)}$, a city-day-location specific fixed effect, and $\delta_{t,l}^{\left(L\right)}$, a location-time-specific fixed effect. For instance, in city *j*, the number of people at work in an office building co-located with a shopping mall will be captured by $\gamma_{j,y,l}^{\left(L\right)}+\delta_{t,l}^{\left(L\right)}$ with *l* being a shopping mall, *y* being a working day, and *t* from 9:00 to 17:00. In other words, our data provides incorrect measures of the number of people at each location type in levels, but may still capture its changes accurately.

Third, it is plausible that some aspects of the measurement errors can potentially be affected by air quality. For instance, some employees may take a day off work if they see a report for poor air quality or if they fall ill due to air pollution; in this case, we will *over-estimate* the negative effects of air pollution on the visits to shopping malls if there are many office buildings co-located with or near a mall. Plausibly, office buildings may have better air filters than many homes in China. For example, [43] used data from six office buildings in China and showed that every 1 *μg*/*m*^3^ increase in outdoor PM_2.5_ is associated with a 0.60 *μg*/*m*^3^ increase in indoor PM_2.5_. In contrast, [44] found that for residential buildings in China, the indoor and outdoor 24-hour averaged PM_2.5_ concentrations were similar; and [45] found that for apartments with normal airtightness and without any HVAC-filter system, indoor and outdoor PM_2.5_ concentrations were significantly correlated, while closed windows could barely help. Therefore, we conjecture that taking a day off due to air pollution might be uncommon. Also, as our sample period covers a single peak of air pollution (Fig 2), people falling ill due to air quality might not have been widespread.

Additionally, if many people go to a shopping mall because of severe air pollution, their phones may have to be connected to far away towers due to the capacity constraints of those at the mall. In this case, we would mistakenly underestimate the number of people at a mall. However, our results indicate that there are insignificantly *fewer* people at shopping malls, making this issue less plausible.

Given these considerations and that our estimates of the effects on the number of individuals at shopping malls (see Table 3) are statistically insignificant, it might be plausible that the true effects are close to zero. Our estimation of the effects on being at home and distance from home are not biased by such measurement errors. Anyone who chooses to skip work to stay at home will be correctly measured as being at home.

Separately, some individuals may go to a park that is less than 500 meters away from their home. We will identify them as at home even when they are at parks. If such people stop going to a park when air pollution is severe, our estimation will understate the effect of air pollution on park visits. Moreover, some phones at a park may need to be connected to some faraway towers when there are too many people at the park. This is more likely to happen when air pollution is low, because air pollution tends to discourage people from going to a park. If it is the case, our results will again underestimate the effect of air pollution. Both underestimations may be partially mitigated by a phone’s frequent switching between towers whenever it moves.

Regarding the analysis of the distance from home, the dependent variable in Eqs (6) and (7) is also measured with error because of the potential imprecision in our geolocating procedure. These measurement errors increase the variance of the error term, *ε*_*i*,*j*,*y*,*t*_, in Eq (6) and thus lower the precision of our estimates. However, to the extent that the measurement errors are random and that *ε*_*i*,*j*,*y*,*t*_ can be assumed to be normally distributed, our estimator of the effect of air pollution on the distance from home remains consistent.

### Potential biases when a user has multiple mobile phones

A user may have multiple mobile phones. S1 Table in S1 File shows that the average penetration rate of mobile phone service ranges from 125% to 212% across the six cities.

Recall that a city’s penetration rate is the total number of subscriptions divided by the city’s population reported in the statistical yearbook. Therefore, the rates in S1 Table in S1 File tend to overestimate the real rates because these six cities are net receivers of migrants from other parts of China. The total population reported in the Statistical Yearbook of Zhejiang in 2014 only includes residents who either had a local residence permit (known as *hukou*) or a temporary residence permit. Migrants from other parts of China who live in one of the six cities without any local residence permit can still subscribe to local mobile service but would not have been counted in the official total number of residents. Moreover, our dataset comes from the largest mobile telecommunication provider in China. When a user has multiple phone lines, he or she might subscribe to services from multiple providers for reasons such as hedging against the risk of service interruptions.

Nonetheless, we can test the robustness of our results by assuming that on average, each user has 1.5 or 2 phones in our dataset. We further impose that the number of phones a user has is *independent* of the user’s daily activity patterns and that each user carries *all* of their phones at all times. We discuss relaxations of these assumptions in the next section on the representativeness of our data.

First, under these assumptions, the location choice results remain the same as before. To see why, note that each of the dependent variables in the regression model, Eq (2), is the difference between two logarithms, ln(*n*_*j*,*y*,*t*,*l*_) − ln(*n*_*j*,*y*,*t*,*h*_), where *n*_*j*,*y*,*t*,*l*_ is the total number of mobile phone users at location *l* (*l* ∈ {*m*, *p*, *o*}) in city *j* on day *y* at time *t*. If every user has *k* phones (*k* = 1.5 or 2) on average, the dependent variable satisfies

$$\begin{array}{c} \ln\left(n_{j,y,t,l}/k\right)-\ln\left(n_{j,y,t,h}/k\right)=\ln\left(n_{j,y,t,l}\right)-\ln\left(n_{j,y,t,h}\right), \end{array}$$

which remains constant. In fact, the above equality holds for any positive *k*.

Second, the distance analysis may change when we account for multiple phones per user. When a user on average has *k* mobile phones in our dataset, dropping (*k* − 1)/*k* observations/phones at each given distance leads to a new sample in which on average every user only has one phone. Because of the large sample size, this drop in data does not change the results much. Specifically, assuming that a user on average has 1.5 phones, S11 and S13 Tables in S1 File show the estimation of the distance equation (Eq 7) with air quality dummies and the AQI as a continuous measure, respectively. Furthermore, when we allowed a user to have two phones on average, we again found very similar results (S12 and S14 Tables in S1 File).

### Representativeness of the data

Three concerns are associated with the representativeness of our data. First, our data may not represent the mobile phone users in these six cities; second, the mobile phone users may not represent the population in these six cities; and third, these six cities may be very different from other cities in China.

The first concern is related to our assumption that a user’s number of phones and his/her daily activities are independent. Active labor market participants and wealthier individuals may be more likely to have multiple phones. Additionally, the users in our sample may be more active in the labor market and wealthier than subscribers of other mobile service providers because our data were drawn from the biggest and oldest provider, which has a wider and more reliable network. Therefore, it is plausible that the users in our sample are less responsive to air pollution; thus, our estimates would provide a lower bound of air pollution’s effects. While some mobile phone users may leave one of their phones at home if they have multiple, it is less likely that this behavior is affected by changes in air pollution (also see the earlier discussion about regulations when air pollution is severe).

Regarding the second concern, mobile phone users may tend to be labor force participants and are less likely to be children or elders. Understandably, children and elders might respond to air pollution more significantly, as often advised in the official guidelines (Table 1). If children and elders are under-represented in our sample, our estimates provide a lower bound of the effects of air pollution.

For the last concern, because of the significant heterogeneity in geography across China, the six cities are far from representative of the general population in China. Given their geographical locations (Fig 1), our results from these six cities may be informative for cities located across the Yangtze River Delta and south of the Huai River. China has the Huai River Policy, whereby the cities to the south of the Huai River do not provide centralized winter heating. This policy leads to more severe air pollution to the north of the Huai River [2, 3]. By changing indoor temperatures, this policy may introduce significant heterogeneity in people’s activities during winter on the two sides of the Huai River; and Zhejiang Province is to the south of the Huai River. The Yangtze River Delta includes Zhejiang Province, Shanghai, and the southern parts of the Jiangsu and Anhui Provinces, and it covers 99,600 *km*^2^ and is home to over 115 million people as of 2013 (see https://en.wikipedia.org/wiki/Yangtze_Delta for more details). The delta is closely integrated, culturally and economically. Moreover, this area is usually exposed to the same waves of air pollution; see the 2013 report of China’s Ministry of Ecology and Environment (in Chinese) available at http://www.mee.gov.cn/hjzl/sthjzk/zghjzkgb/201605/P020160526564151497131.pdf.

### Other sensitivity analyses

We also perform an additional set of sensitivity analyses. In the analysis of location choice, column (3) of S5 Table in S1 File controls for PM_2.5_ and shows qualitatively similar coefficients on air quality dummies, which may be explained by the low accessibility of PM_2.5_ data. Further, including the lagged air quality dummies leads to nearly identical results (column 4). In the last three columns (columns 5 to 7), we add weather dummies in the regressions and again obtain very similar results. Recall that hourly weather data were collected every three hours. Thus, we have a smaller sample for these three regressions. Columns (3) to (7) of S6 Table in S1 File repeat the same set of exercises for the analysis of location choice with the AQI as a continuous variable. The results remain qualitatively similar.

In the regressions of distance presented in S7 and S8 Tables in S1 File, the same set of sensitivity analyses (columns 3 to 7) of each table also show the robustness of our results.

## Conclusions and discussions

We developed and applied a statistical/econometric model to hourly data of 9 million mobile phone users from six cities in China’s Zhejiang Province to measure the effects of air pollution on human activities. One of the main findings is that many individuals choose to be at home when air pollution worsens. This offers new insights into the design of public policies. For example, the official recommendations (Table 1) state that when the level of air quality is “Heavily Polluted,” “children, senior citizens, and individuals with respiratory or cardiac issues should *stay indoors*.” However, the recommendation of staying indoors may not be sufficient because indoor air quality could be poor due to cigarette smoking, home cooking, chemicals from decorations, or chemical air pollutants from outdoor. Moreover, an individual’s home may not be well insulated against outdoor pollutants [44, 45]. On the other hand, shopping malls, museums, schools, and other public indoor facilities in China have started to take measures to clean the indoor air [25, 26, 43]. Therefore, a better recommendation might be to ask individuals to stay at an indoor facility (their home or somewhere else) with clean air. Besides, encouraging households to reduce indoor air pollution and better insulate their homes when air pollution is severe would also be beneficial.

Our results also highlight the urgent need to monitor indoor air quality. Although outdoor air quality is reported regularly, the air quality at home or the majority of indoor facilities is often unmeasured. A city government may work with various parties to encourage regular monitoring of indoor air quality.

Due to the limitations of our data, several caveats are in order. First and foremost, our measurement of individuals’ location is coarse and contains errors. We can only identify one’s home, some parks, and some shopping malls, but not their workplace. In the geolocation procedure, some individuals who are close to a park may be counted as being at the park; some who are actually at a park may not be identified as at the park. All these issues are because our data are mobile phone logs. Our modeling is shown to be able to address some of these issues, but potential biases in the estimation may still exist. If one has access to the precise GPS location of a phone, these issues can be better addressed. Second, our data only contain four days and do not allow us to build a time series of each individual’s activities. It prevents us from precisely estimating intra-day substitutions of activities and heterogeneous effects of air pollution across weekends and weekdays. Lastly, our data do not have any demographic or socioeconomic characteristics of the phone users, which makes it impossible to investigate heterogeneous responses across different groups of individuals. Since children and elderlies are considered to be more sensitive to air pollution, it is important to investigate their behavioral responses to air pollution. Collecting more and better data for future research will be fruitful.

## Supporting information

S1 File (PDF)
