# Supplementary material for: Impact of air pollution on human activities: Evidence from nine million mobile phone users
Source: PLoS One. 2021 May 19;16(5):e0251288. doi: 10.1371/journal.pone.0251288 (PMC8133462; doi:10.1371/journal.pone.0251288)
Supplement: S1 File — (PDF) [file pone.0251288.s001.pdf]

Four sets of additional results are included to provide additional details about the study.

The first set includes two figures and four tables of summary statistics. S1 Fig depicts the hourly reported $PM_{2.5}$ and PM_{10}. S1 Table provides characteristics of the six cities in our data. S2 Table shows the number of identified parks and shopping malls in each city. S3 Table presents the summary statistics of the full sample and those of the weather subsample. S4 Table describes the weather data. S2 Fig does a sanity check our location data by comparing individuals' location choice on Saturday with that on other days in the sample.

The second set of tables are about estimation results. S5 Table and S6 Table present the results of the location choice analyses with air quality dummies and AQI, respectively. Similarly, S7 Table and S8 Table provide the results from the distance analyses. Each table includes the benchmark specifications, columns (1) and (2), as well as robustness checks, columns (3)–(7).

The third set of materials are two tables on analyses with daily data—S9 Table on location choice and S10 Table on distance from home.

The last set of tables present our robustness checks when we consider multiple phones per user in the analysis of distance from home. Specifically, we first assume that, on average, an individual has 1.5 mobile phones, with air quality dummies (S11 Table) or AQI as a continuous variable (S13 Table). We then assume that every individual in the data has 2 mobile phones (S12 Table and S14 Table). The estimation results are almost identical to those in S7 Table and S8 Table where we assume that a mobile phone corresponds to a unique user.

(a) $PM_{2.5}$ (b) PM_{10}

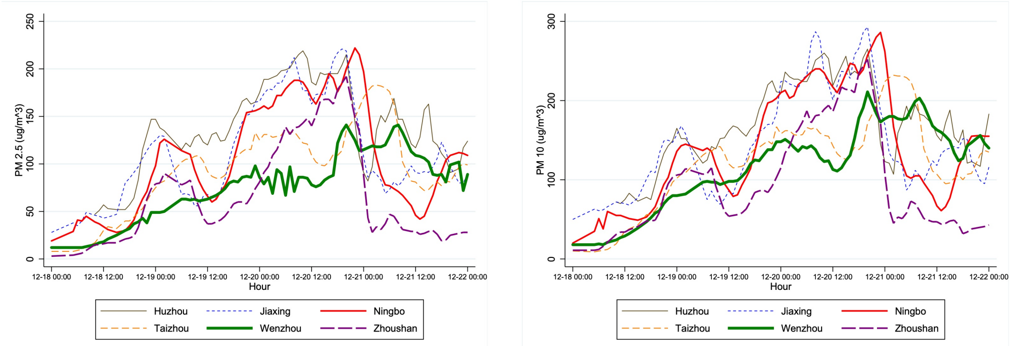

S1 Fig. Hourly Reported $PM_{2.5}$ and PM_{10} ($\mu g/m^3$) in the Six Cities from December 18 to 21, 2013

S1 Table. City Characteristics and Mobile Phone Service in 2013

City	Surface Area (km^2) (1)	GPD per capita (Yuan) (2)	Population (million) (3)	Mobile Phone Service: Total Subscriptions (million) (4)	Mobile Phone Service: Penetration Rate (%) (5)
Huzhou	5,824	65,871	2.62	3.66	140
Jiaxing	3,915	91,177	3.46	5.61	162
Ningbo	9,845	123,139	5.80	12.28	212
Taizhou	9,411	53,222	5.94	7.45	125
Wenzhou	11,784	49,817	8.07	11.85	147
Zhoushan	1,455	95,726	0.97	1.57	161

Notes: All data are year-end in 2013. On December 31, 2013, one U.S. dollar was about 6.054 Chinese yuan. The penetration rate is the average number of mobile phone subscriptions per capita (column 4 divided by column 3). Source: Statistical Yearbook of Zhejiang, 2014.

S2 Table. Numbers of Identified Parks and Shopping Malls Across the Cities

	Huzhou	Jiaxing	Ningbo	Taizhou	Wenzhou	Zhoushan	Total
Park	13	7	15	7	19	7	68
Shopping mall	4	6	9	6	11	4	40

S3 Table. Summary Statistics: Full Sample and Weather Subsample

Variable	# Observations	Mean	Std. Dev.	Min	Max
Panel A. Full sample					
AQI	384	118.17	62.76	19	245
$PM_{2.5}$	381	95.20	54.18	6	222
Air quality dummies:					
$Excellent_{j,y,t}$	384	0.18	0.38	0	1
$Good_{j,y,t}$	384	0.21	0.41	0	1
$Slightly\_Polluted_{j,y,t}$	384	0.29	0.46	0	1
$Moderately\_Polluted_{j,y,t}$	384	0.18	0.38	0	1
$Heavily\_Polluted_{j,y,t}$	384	0.14	0.34	0	1
Log odds ratio (multiplied by 1000):					
$\ln(n_{j,y,t,o}) - \ln(n_{j,y,t,h})$ (other vs. home)	384	708.48	419.57	-367.86	1450.99
$\ln(n_{j,y,t,p}) - \ln(n_{j,y,t,h})$ (park vs. home)	384	-3665.60	685.03	-5563.31	-2328.53
$\ln(n_{j,y,t,m}) - \ln(n_{j,y,t,h})$ (mall vs. home)	384	-4415.75	802.33	-6757.54	-2823.74
Panel B. Weather subsample					
AQI	144	118.33	63.09	19	245
$PM_{2.5}$	143	95.82	53.87	6	222
Air quality dummies:					
$Excellent_{j,y,t}$	144	0.18	0.39	0	1
$Good_{j,y,t}$	144	0.22	0.42	0	1
$Slightly\_Polluted_{j,y,t}$	144	0.28	0.45	0	1
$Moderately\_Polluted_{j,y,t}$	144	0.18	0.39	0	1
$Heavily\_Polluted_{j,y,t}$	144	0.14	0.35	0	1
Log odds ratio (multiplied by 1000):					
$\ln(n_{j,y,t,o}) - \ln(n_{j,y,t,h})$ (other vs. home)	144	612.64	481.98	-367.86	1414.74
$\ln(n_{j,y,t,p}) - \ln(n_{j,y,t,h})$ (park vs. home)	144	-3795.75	757.20	-5563.31	-2358.61
$\ln(n_{j,y,t,m}) - \ln(n_{j,y,t,h})$ (mall vs. home)	144	-4576.10	933.82	-6757.54	-2823.74

Notes: For each log odds ratio for a given location l (l is others, park, or mall) versus home, it is calculated for city j, day y, at time t ($t = 7, 8, \ldots, 22$). There are 6 cities, 4 days, and 16 hours per day, and therefore, we obtain the number of observations, 384, in the full sample. There are only 144 observations in the weather subsample because weather data is reported every three hours. Each dummy variable for air quality equals one if the air quality in city j at hour t of day y is at that level and zero otherwise. There is no observation that the air is "Severely Polluted." The AQI range for each air quality level is shown in Table 1.

S4 Table. Summary Statistics of Weather Data

	Freq.	Percent	Cum.
Panel A. Weather code			
No weather phenomena to report	46	31.94	31.94
Smog	82	56.94	88.89
Light fog	12	8.33	97.22
Rain	2	1.39	98.61
Shower	2	1.39	100
Total	144	100	
Panel B. Wind speed code			
0: 0 meter per second	3	2.08	2.08
1: 1 meter per second	7	4.86	6.94
2: 2 meters per second	27	18.75	25.69
3: 4 meters per second	38	26.39	52.08
4: 7 meters per second	37	25.69	77.78
5: 9 meters per second	16	11.11	88.89
6: 12 meters per second	8	5.56	94.44
7: 16 meters per second	5	3.47	97.92
8: 19 meters per second	3	2.08	100
Total	144	100	

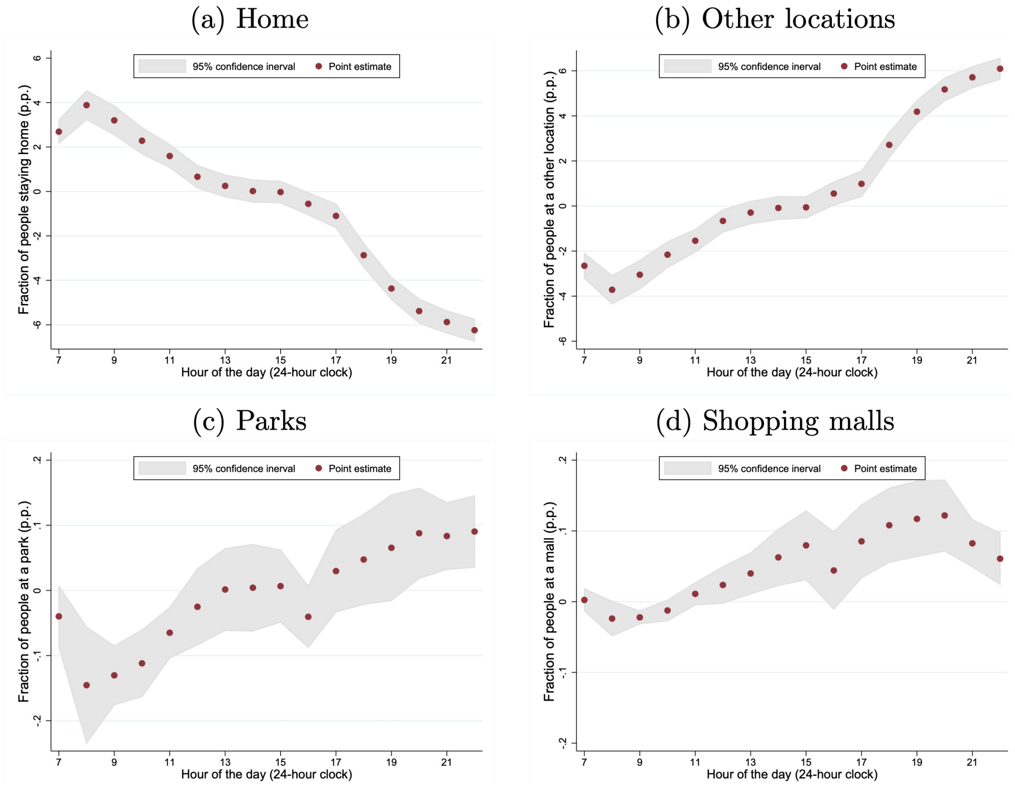

S2 Fig. Location Choices in Each Hour on Saturday vs. on Weekdays

Notes: Panel (a) show the estimated coefficients and their 95% confidence intervals from a regression of the fraction of people at home, in percentage points, on a dummy variable for Saturday interacted with 16 dummies for the hours of the day from 7:00 to 22:00 while controlling for city-hour fixed effects. The regression uses hourly data for the six cities from December 18 to 21 (Wednesday to Saturday), 2013, and only includes the hours from 7:00 to 22:00 on each day. Panels (b)–(d) are similarly obtained, except that the dependent variables are the fractions of people (in percentage points) at other locations, parks, and shopping malls, respectively.

S5 Table. Estimation Results: Location Choice with Air Quality Categories

	Full Sample				Weather Subsample		
	(1)	(2)	(3)	(4)	(5)	(6)	(7)
				Panel A. Other Locations			
$Heavily\_Polluted_{j,y,t}$	-278.095***	-482.959***	-255.088***		-279.742***	-441.444***	-265.017***
	(31.541)	(46.983)	(25.977)		(65.213)	(72.468)	(56.236)
$t \times Heavily\_Polluted_{j,y,t}$		53.160***				36.091*	
		(11.667)				(18.256)	
$t^2 \times Heavily\_Polluted_{j,y,t}$		-2.752***				-1.956	
		(0.782)				(1.222)	
$Moderately\_Polluted_{j,y,t}$	-136.954***	-355.604***	-136.457***		-140.480***	-400.841***	-138.272***
	(22.907)	(36.929)	(21.224)		(46.656)	(58.855)	(42.169)
$t \times Moderately\_Polluted_{j,y,t}$		44.318***				51.438***	
		(9.450)				(15.645)	
$t^2 \times Moderately\_Polluted_{j,y,t}$		-1.405**				-1.832*	
		(0.620)				(1.051)	
$Slightly\_Polluted_{j,y,t}$	-90.115***	-143.599***	-71.427***		-102.387**	-147.244***	-84.889**
	(18.821)	(32.902)	(14.976)		(40.701)	(55.313)	(36.175)
$t \times Slightly\_Polluted_{j,y,t}$		24.281**				23.785	
		(9.977)				(15.208)	
$t^2 \times Slightly\_Polluted_{j,y,t}$		-1.384**				-1.446	
		(0.691)				(1.017)	
$Excellent_{j,y,t}$	59.241*	-20.071	1.222		45.313	-1.841	-23.200
	(31.307)	(47.017)	(28.630)		(49.225)	(69.158)	(40.282)
$t \times Excellent_{j,y,t}$		8.321				8.086	
		(9.191)				(15.786)	
$t^2 \times Excellent_{j,y,t}$		-0.804				-0.921	
		(0.641)				(1.028)	
$PM_{2.5j,y,t}$			-2.135***				-1.910***
			(0.275)				(0.465)
$Heavily\_Polluted_{j,y,t-1}$ (lag)				-268.567***			
				(27.984)			
$Moderately\_Polluted_{j,y,t-1}$ (lag)				-130.140***			
				(21.581)			
$Slightly\_Polluted_{j,y,t-1}$ (lag)				-93.534***			
				(17.179)			
$Excellent_{j,y,t-1}$ (lag)				76.867**			
				(31.912)			
Weather Controls	No	No	No	No	Yes	Yes	Yes
N	384	384	381	384	144	144	143
R^2	0.970	0.979	0.976	0.970	0.978	0.984	0.982

S5 Table. (*Continued*)

	Full Sample				Weather Subsample		
	(1)	(2)	(3)	(4)	(5)	(6)	(7)
				Panel B. Park			
$Heavily\_Polluted_{j,y,t}$	-359.907***	-645.299***	-329.485***		-340.963***	-561.122***	-324.770***
	(50.804)	(102.789)	(43.915)		(101.975)	(149.369)	(90.191)
$t \times Heavily\_Polluted_{j,y,t}$		68.501***				42.028	
		(23.622)				(33.564)	
$t^2 \times Heavily\_Polluted_{j,y,t}$		-3.317**				-1.934	
		(1.405)				(2.016)	
$Moderately\_Polluted_{j,y,t}$	-151.409***	-378.657***	-148.495***		-176.989***	-450.896***	-172.929***
	(32.959)	(62.954)	(33.645)		(62.479)	(106.634)	(63.419)
$t \times Moderately\_Polluted_{j,y,t}$		39.002**				46.395	
		(17.289)				(29.738)	
$t^2 \times Moderately\_Polluted_{j,y,t}$		-0.838				-1.353	
		(1.013)				(1.764)	
$Slightly\_Polluted_{j,y,t}$	-123.286***	-126.581**	-98.365***		-141.584***	-166.184*	-125.016***
	(24.715)	(52.591)	(21.440)		(46.927)	(85.821)	(46.619)
$t \times Slightly\_Polluted_{j,y,t}$		11.149				15.237	
		(17.567)				(27.362)	
$t^2 \times Slightly\_Polluted_{j,y,t}$		-0.847				-1.062	
		(1.037)				(1.529)	
$Excellent_{j,y,t}$	122.657**	73.184	47.113		102.983	111.680	12.497
	(51.879)	(86.398)	(51.956)		(78.046)	(134.960)	(75.086)
$t \times Excellent_{j,y,t}$		-1.349				-25.500	
		(17.426)				(25.803)	
$t^2 \times Excellent_{j,y,t}$		-0.557				0.317	
		(1.046)				(1.483)	
$PM_{2.5j,y,t}$			-2.651***				-2.246***
			(0.433)				(0.793)
$Heavily\_Polluted_{j,y,t-1}$ (lag)				-335.938***			
				(43.215)			
$Moderately\_Polluted_{j,y,t-1}$ (lag)				-139.302***			
				(31.473)			
$Slightly\_Polluted_{j,y,t-1}$ (lag)				-122.024***			
				(22.329)			
$Excellent_{j,y,t-1}$ (lag)				140.891***			
				(49.474)			
Weather Controls	No	No	No	No	Yes	Yes	Yes
N	384	384	381	384	144	144	143
R^2	0.973	0.980	0.977	0.973	0.978	0.983	0.981

S5 Table. (*Continued*)

	Full Sample				Weather Subsample		
	(1)	(2)	(3)	(4)	(5)	(6)	(7)
				Panel C. Shopping Mall			
$Heavily\_Polluted_{j,y,t}$	-357.764***	-869.323***	-320.635***		-248.694	-566.812**	-231.225
	(98.742)	(174.749)	(94.944)		(185.034)	(273.405)	(179.827)
$t \times Heavily\_Polluted_{j,y,t}$		144.040***				108.466*	
		(39.421)				(56.247)	
$t^2 \times Heavily\_Polluted_{j,y,t}$		-6.910***				-5.735**	
		(2.103)				(2.819)	
$Moderately\_Polluted_{j,y,t}$	-169.387**	-458.189***	-162.684**		-77.444	-297.556	-69.911
	(75.106)	(110.675)	(71.894)		(160.413)	(241.613)	(152.455)
$t \times Moderately\_Polluted_{j,y,t}$		80.424**				57.461	
		(31.263)				(58.281)	
$t^2 \times Moderately\_Polluted_{j,y,t}$		-3.387**				-2.469	
		(1.695)				(2.933)	
$Slightly\_Polluted_{j,y,t}$	-119.431*	-194.119*	-88.430		-116.163	-116.068	-103.462
	(65.389)	(113.599)	(59.716)		(144.420)	(211.308)	(132.667)
$t \times Slightly\_Polluted_{j,y,t}$		37.736				21.008	
		(33.832)				(53.870)	
$t^2 \times Slightly\_Polluted_{j,y,t}$		-2.072				-1.554	
		(1.883)				(2.849)	
$Excellent_{j,y,t}$	188.222***	41.334	97.633***		197.855	151.749	70.950
	(48.486)	(131.920)	(37.186)		(179.669)	(238.803)	(132.497)
$t \times Excellent_{j,y,t}$		8.439				-11.843	
		(35.345)				(50.942)	
$t^2 \times Excellent_{j,y,t}$		-0.876				-0.142	
		(1.947)				(2.647)	
$PM_{2.5\,j,y,t}$			-3.020***				-2.704***
			(0.582)				(0.994)
$Heavily\_Polluted_{j,y,t-1}$ (lag)				-332.713***			
				(85.745)			
$Moderately\_Polluted_{j,y,t-1}$ (lag)				-158.683**			
				(66.310)			
$Slightly\_Polluted_{j,y,t-1}$ (lag)				-116.101**			
				(54.823)			
$Excellent_{j,y,t-1}$ (lag)				193.884***			
				(47.056)			
Weather Controls	No	No	No	No	Yes	Yes	Yes
N	384	384	381	384	144	144	143
R^2	0.962	0.972	0.967	0.962	0.967	0.972	0.973

Notes: This table presents the estimates of Eq (2), with the dependent variable being the log odds ratio (multiplied by 1000). An observation is for city j on day y in hour t. There are 6 cities, 4 days, and 16 hours per day, leading to 384 observations in total. Some regressions have fewer than 384 observations because there are missing values in $PM_{2.5}$ and weather variables. Each column in a panel is a regression, and every regression includes fixed effects $\gamma^{(L)}_{j,y,l}$ (average popularity of location l in city j on day y) and $\delta^{(L)}_{t,l}$ (average popularity of location l at time t). Columns (5)–(7) also include two sets of dummy variables on weather—one set captures the weather categories in Panel A of S4 Table; the other captures the wind speed categories in Panel B of S4 Table. Heteroskedasticity robust standard errors are in parentheses. * $p < 0.10$, ** $p < 0.05$, *** $p < 0.01$.

S6 Table. Estimation Results: Location Choice with AQI as a Continuous Measure

	Full Sample				Weather Subsample		
	(1)	(2)	(3)	(4)	(5)	(6)	(7)
				Panel A. Other Locations			
$AQI_{j,y,t}$	-3.167***	-3.947***	-2.786***		-2.863***	-3.451***	-2.594***
	(0.291)	(0.313)	(0.248)		(0.544)	(0.548)	(0.486)
$t \times AQI_{j,y,t}$		0.213***				0.173**	
		(0.043)				(0.076)	
$t^2 \times AQI_{j,y,t}$		-0.008***				-0.007	
		(0.003)				(0.005)	
$PM_{2.5j,y,t}$			-1.729***				-1.528***
			(0.241)				(0.402)
$AQI_{j,y,t-1}$ (lag)				-3.045***			
				(0.289)			
Weather Controls	No	No	No	No	Yes	Yes	Yes
N	384	384	381	384	144	144	143
R^2	0.975	0.980	0.980	0.974	0.982	0.983	0.985
				Panel B. Park			
$AQI_{j,y,t}$	-3.744***	-4.922***	-3.282***		-2.888***	-4.016***	-2.634***
	(0.479)	(0.591)	(0.420)		(0.854)	(1.004)	(0.764)
$t \times AQI_{j,y,t}$		0.294***				0.293**	
		(0.093)				(0.144)	
$t^2 \times AQI_{j,y,t}$		-0.010*				-0.009	
		(0.006)				(0.009)	
$PM_{2.5j,y,t}$			-2.304***				-1.950***
			(0.404)				(0.732)
$AQI_{j,y,t-1}$ (lag)				-3.581***			
				(0.459)			
Weather Controls	No	No	No	No	Yes	Yes	Yes
N	384	384	381	384	144	144	143
R^2	0.975	0.979	0.978	0.974	0.978	0.981	0.981
				Panel C. Shopping Mall			
$AQI_{j,y,t}$	-3.421***	-5.422***	-2.932***		-2.287	-3.803**	-2.089
	(0.793)	(0.907)	(0.802)		(1.487)	(1.677)	(1.495)
$t \times AQI_{j,y,t}$		0.627***				0.591**	
		(0.146)				(0.235)	
$t^2 \times AQI_{j,y,t}$		-0.028***				-0.029**	
		(0.008)				(0.012)	
$PM_{2.5j,y,t}$			-2.717***				-2.633***
			(0.555)				(0.968)
$AQI_{j,y,t-1}$ (lag)				-3.217***			
				(0.775)			
Weather Controls	No	No	No	No	Yes	Yes	Yes
N	384	384	381	384	144	144	143
R^2	0.963	0.970	0.968	0.962	0.967	0.970	0.973

Notes: This table presents the estimates of Eq (2), with the dependent variable being the log odds ratio (multiplied by 1000). An observation is for city j on day y in hour t. There are 6 cities, 4 days, and 16 hours per day, leading to 384 observations. Some regressions have fewer than 384 observations because there are missing values in $PM_{2.5}$ and weather variables. Each column in a panel is a regression, and every regression includes fixed effects $\gamma^{(L)}_{j,y,l}$ (average popularity of location l in city j on day y) and $\delta^{(L)}_{t,l}$ (average popularity of location l at time t). Columns (5)–(7) also include two sets of dummy variables on weather conditions—one set captures the weather categories in Panel A of S4 Table; the other captures the wind speed categories in Panel B of S4 Table. Heteroskedasticity robust standard errors are in parentheses. * $p < 0.10$, ** $p < 0.05$, *** $p < 0.01$.

S7 Table. Estimation Results: Distance from Home with Air Quality Categories

	Full Sample				Weather Subsample		
	(1)	(2)	(3)	(4)	(5)	(6)	(7)
$Heavily\_Polluted_{j,y,t}$	-635.313***	-868.875***	-713.685***		-674.049***	-742.127***	-681.970***
	(53.021)	(77.939)	(64.465)		(126.489)	(134.860)	(115.031)
$t \times Heavily\_Polluted_{j,y,t}$		94.895***				54.733**	
		(17.677)				(26.281)	
$t^2 \times Heavily\_Polluted_{j,y,t}$		-6.124***				-4.087***	
		(1.127)				(1.566)	
$Moderately\_Polluted_{j,y,t}$	-358.129***	-721.294***	-470.454***		-415.176***	-860.142***	-435.368***
	(45.365)	(70.135)	(58.466)		(113.372)	(110.113)	(103.739)
$t \times Moderately\_Polluted_{j,y,t}$		86.071***				122.549***	
		(14.799)				(22.295)	
$t^2 \times Moderately\_Polluted_{j,y,t}$		-2.666***				-5.225***	
		(1.023)				(1.535)	
$Slightly\_Polluted_{j,y,t}$	-207.779***	-307.793***	-227.968***		-253.380**	-337.145**	-206.375**
	(41.452)	(86.990)	(49.616)		(102.546)	(132.522)	(92.379)
$t \times Slightly\_Polluted_{j,y,t}$		37.770*				43.076	
		(22.277)				(28.310)	
$t^2 \times Slightly\_Polluted_{j,y,t}$		-1.553				-1.848	
		(1.613)				(1.969)	
$Excellent_{j,y,t}$	220.830***	112.701	47.921		222.425*	171.591	49.597
	(58.692)	(76.082)	(50.945)		(117.988)	(114.818)	(71.965)
$t \times Excellent_{j,y,t}$		-0.185				0.679	
		(17.695)				(22.696)	
$t^2 \times Excellent_{j,y,t}$		-0.924				-1.289	
		(1.341)				(1.541)	
$PM_{2.5j,y,t}$			-6.827***				-5.794***
			(0.784)				(1.050)
$Heavily\_Polluted_{j,y,t-1}$ (lag)				-599.360***			
				(51.116)			
$Moderately\_Polluted_{j,y,t-1}$ (lag)				-312.860***			
				(44.967)			
$Slightly\_Polluted_{j,y,t-1}$ (lag)				-182.513***			
				(41.693)			
$Excellent_{j,y,t-1}$ (lag)				251.649***			
				(57.616)			
σ	6359.209***	6358.744***	6359.345***	6359.177***	6294.060***	6293.561***	6295.513***
	(56.583)	(56.575)	(56.739)	(56.581)	(95.199)	(95.197)	(95.395)
Weather Controls	No	No	No	No	Yes	Yes	Yes
N	579,876,533	579,876,533	578,007,769	579,876,533	217,449,349	217,449,349	216,827,193

Notes: This table presents the estimates of Eq (6), with the dependent variable being the distance from home. An observation is an individual in city j on day y in hour t. Each column is a regression, and every regression includes fixed effects $\gamma_{j,y}^{(D)}$ (average willingness to travel in city j on day y) and $\delta_t^{(D)}$ (average willingness to travel at time t). Columns (5)–(7) also include two sets of dummy variables on weather conditions—one set captures the weather categories in Panel A of S4 Table; the other captures the wind speed categories in Panel B of S4 Table. Heteroskedasticity robust standard errors are in parentheses. * $p < 0.10$, ** $p < 0.05$, *** $p < 0.01$.

S8 Table. Estimation Results: Distance from Home with AQI as a Continuous Measure

	Full Sample				Weather Subsample		
	(1)	(2)	(3)	(4)	(5)	(6)	(7)
$AQI_{j,y,t}$	-7.907***	-9.146***	-7.424***		-7.594***	-8.180***	-7.168***
	(0.650)	(0.720)	(0.510)		(0.836)	(0.883)	(0.702)
$t \times AQI_{j,y,t}$		0.537***				0.389***	
		(0.101)				(0.145)	
$t^2 \times AQI_{j,y,t}$		-0.028***				-0.020**	
		(0.007)				(0.010)	
$PM_{2.5j,y,t}$			-5.946***				-5.023***
			(0.701)				(0.928)
$AQI_{j,y,t-1}$ (lag)				-7.467***			
				(0.605)			
σ	6358.836***	6358.674***	6359.105***	6358.917***	6293.693***	6293.602***	6295.241***
	(56.584)	(56.590)	(56.736)	(56.583)	(95.204)	(95.214)	(95.394)
Weather Controls	No	No	No	No	Yes	Yes	Yes
N	579,876,533	579,876,533	578,007,769	579,876,533	217,449,349	217,449,349	216,827,193

Notes: This table presents the estimates of Eq (6), with the dependent variable being the distance from home. An observation is an individual in city j on day y in hour t. Each column is a regression, and every regression includes fixed effects $\gamma_{j,y}^{(D)}$ (average willingness to travel in city j on day y) and $\delta_t^{(D)}$ (average willingness to travel at time t). Columns (5)–(7) also include two sets of dummy variables on weather—one set captures the weather categories in Panel A of S4 Table; the other captures the wind speed categories in Panel B of S4 Table. Heteroskedasticity robust standard errors are in parentheses. * $p < 0.10$, ** $p < 0.05$, *** $p < 0.01$.

S9 Table. Analysis of Daily Data—Location Choice

	Home (1)	Park (2)	Shopping Mall (3)	Other (4)
AQI: Daily max (during 7:00–22:00)	0.050	-0.003	-0.001	-0.047
	(0.049)	(0.003)	(0.003)	(0.050)
AQI: Daily mean (during 7:00–22:00)	0.047	-0.004	-0.001	-0.042
	(0.051)	(0.003)	(0.003)	(0.052)
AQI: Daily median (during 7:00–22:00)	0.041	-0.003	-0.001	-0.037
	(0.050)	(0.003)	(0.003)	(0.050)
AQI: Daily officially reported value	0.091	-0.006*	-0.001	-0.083
	(0.054)	(0.004)	(0.003)	(0.055)
N	24	24	24	24

Notes: Each entry is an estimate from a unique linear regression. The dependent variable is the percentage of individuals being at home, a park, a shopping mall, or other locations in each hour in a given city on a given day, averaged over the 16 hours (7:00–22:00), multiplied by 10. Every regression includes day and city fixed effects. There are 24 observations in every regression. Homoskedastic standard errors are in parentheses. * $p < 0.10$, ** $p < 0.05$, *** $p < 0.01$.

S10 Table. Analysis of Daily Data—Distance from Home

AQI:	Daily max (1)	Daily mean (2)	Daily median (3)	Daily officially reported value (4)
Coefficient	-1.775	-2.680*	-2.687*	-3.417**
Standard error	(1.512)	(1.460)	(1.404)	(1.585)
R^2	0.943	0.950	0.950	0.953
N	24	24	24	24

Notes: Each column presents results from a regression, with the dependent variable being the daily average distance for city j on day y, $\frac{1}{16}\sum_{t=7}^{22}\frac{1}{n_{j,y,t}}\sum_{i=1}^{n_j} d_{i,j,y,t}$. Every regression includes day and city fixed effects. There are 24 observations in every regression. Homoskedastic standard errors are in parentheses. * $p < 0.10$, ** $p < 0.05$, *** $p < 0.01$.

S11 Table. Robustness of Results on Distance with Air Quality Categories: 1.5 Phones per User

	Full Sample				Weather Subsample		
	(1)	(2)	(3)	(4)	(5)	(6)	(7)
$Heavily\_Polluted_{j,y,t}$	-635.460***	-868.868***	-713.839***		-673.981***	-741.829***	-681.927***
	(52.996)	(77.934)	(64.452)		(126.405)	(134.790)	(114.956)
$t \times Heavily\_Polluted_{j,y,t}$		94.847***				54.712**	
		(17.684)				(26.281)	
$t^2 \times Heavily\_Polluted_{j,y,t}$		-6.121***				-4.086***	
		(1.126)				(1.566)	
$Moderately\_Polluted_{j,y,t}$	-358.342***	-721.322***	-470.655***		-415.326***	-860.038***	-435.515***
	(45.354)	(70.104)	(58.467)		(113.308)	(110.026)	(103.689)
$t \times Moderately\_Polluted_{j,y,t}$		86.029***				122.530***	
		(14.804)				(22.290)	
$t^2 \times Moderately\_Polluted_{j,y,t}$		-2.665***				-5.226***	
		(1.023)				(1.534)	
$Slightly\_Polluted_{j,y,t}$	-207.978***	-307.918***	-228.179***		-253.555**	-337.220**	-206.590**
	(41.449)	(86.949)	(49.619)		(102.498)	(132.452)	(92.328)
$t \times Slightly\_Polluted_{j,y,t}$		37.711*				43.020	
		(22.275)				(28.304)	
$t^2 \times Slightly\_Polluted_{j,y,t}$		-1.549				-1.844	
		(1.613)				(1.968)	
$Excellent_{j,y,t}$	220.800***	112.922	47.946		222.294*	171.558	49.535
	(58.727)	(76.164)	(51.023)		(117.825)	(114.709)	(71.965)
$t \times Excellent_{j,y,t}$		-0.283				0.607	
		(17.703)				(22.693)	
$t^2 \times Excellent_{j,y,t}$		-0.918				-1.283	
		(1.342)				(1.540)	
$PM_{2.5 j,y,t}$			-6.825***				-5.791***
			(0.784)				(1.050)
$Heavily\_Polluted_{j,y,t-1}$ (lag)				-599.434***			
				(51.098)			
$Moderately\_Polluted_{j,y,t-1}$ (lag)				-313.020***			
				(44.964)			
$Slightly\_Polluted_{j,y,t-1}$ (lag)				-182.643***			
				(41.699)			
$Excellent_{j,y,t-1}$ (lag)				251.539***			
				(57.648)			
σ	6349.089***	6348.623***	6349.274***	6349.057***	6283.532***	6283.032***	6285.041***
	(56.708)	(56.700)	(56.864)	(56.706)	(95.430)	(95.428)	(95.623)
Weather Controls	No	No	No	No	Yes	Yes	Yes
N	386,520,317	386,520,317	385,274,973	386,520,317	144,942,148	144,942,148	144,527,552

Notes: This table presents the estimates of Eq (6), with the dependent variable being the distance from home. An observation is an individual in city j on day y in hour t. Each column is a regression, and every regression includes fixed effects $\gamma_{j,y}^{(D)}$ (average willingness to travel in city j on day y) and $\delta_t^{(D)}$ (average willingness to travel at time t). Columns (5)–(7) also include two sets of dummy variables on weather—one set captures the weather categories in Panel A of S4 Table; the other captures the wind speed categories in Panel B of S4 Table. Heteroskedasticity robust standard errors are in parentheses. * $p < 0.10$, ** $p < 0.05$, *** $p < 0.01$.

S12 Table. Robustness of Results on Distance with Air Quality Categories: 2 Phones per User

	Full Sample				Weather Subsample		
	(1)	(2)	(3)	(4)	(5)	(6)	(7)
$Heavily\_Polluted_{j,y,t}$	-635.214***	-868.683***	-713.603***		-673.556***	-741.566***	-681.484***
	(53.009)	(77.937)	(64.470)		(126.489)	(134.812)	(115.054)
$t \times Heavily\_Polluted_{j,y,t}$		94.874***				54.755**	
		(17.680)				(26.272)	
$t^2 \times Heavily\_Polluted_{j,y,t}$		-6.122***				-4.086***	
		(1.126)				(1.565)	
$Moderately\_Polluted_{j,y,t}$	-358.218***	-721.173***	-470.545***		-415.034***	-859.790***	-435.221***
	(45.360)	(70.124)	(58.475)		(113.372)	(110.075)	(103.757)
$t \times Moderately\_Polluted_{j,y,t}$		86.012***				122.518***	
		(14.809)				(22.293)	
$t^2 \times Moderately\_Polluted_{j,y,t}$		-2.663***				-5.223***	
		(1.024)				(1.535)	
$Slightly\_Polluted_{j,y,t}$	-207.868***	-307.873***	-228.075***		-253.224**	-336.952**	-206.235**
	(41.453)	(86.970)	(49.625)		(102.541)	(132.467)	(92.385)
$t \times Slightly\_Polluted_{j,y,t}$		37.718*				43.012	
		(22.279)				(28.297)	
$t^2 \times Slightly\_Polluted_{j,y,t}$		-1.548				-1.842	
		(1.614)				(1.968)	
$Excellent_{j,y,t}$	220.695***	112.714	47.830		222.378*	171.562	49.619
	(58.707)	(76.116)	(50.939)		(118.043)	(114.784)	(72.003)
$t \times Excellent_{j,y,t}$		-0.280				0.533	
		(17.711)				(22.692)	
$t^2 \times Excellent_{j,y,t}$		-0.917				-1.278	
		(1.342)				(1.540)	
$PM_{2.5j,y,t}$			-6.825***				-5.792***
			(0.784)				(1.051)
$Heavily\_Polluted_{j,y,t-1}$ (lag)				-599.349***			
				(51.103)			
$Moderately\_Polluted_{j,y,t-1}$ (lag)				-312.963***			
				(44.968)			
$Slightly\_Polluted_{j,y,t-1}$ (lag)				-182.671***			
				(41.699)			
$Excellent_{j,y,t-1}$ (lag)				251.535***			
				(57.646)			
σ	6349.097***	6348.631***	6349.282***	6349.065***	6283.584***	6283.084***	6285.086***
	(56.704)	(56.696)	(56.860)	(56.702)	(95.421)	(95.419)	(95.615)
Weather Controls	No	No	No	No	Yes	Yes	Yes
N	289,890,212	289,890,212	288,956,212	289,890,212	108,706,659	108,706,659	108,395,709

Notes: This table presents the estimates of Eq (6), with the dependent variable being the distance from home. An observation is an individual in city j on day y in hour t. Each column is a regression, and every regression includes fixed effects $\gamma_{j,y}^{(D)}$ (average willingness to travel in city j on day y) and $\delta_t^{(D)}$ (average willingness to travel at time t). Columns (5)–(7) also include two sets of dummy variables on weather—one set captures the weather categories in Panel A of S4 Table; the other captures the wind speed categories in Panel B of S4 Table. Heteroskedasticity robust standard errors are in parentheses. * $p < 0.10$, ** $p < 0.05$, *** $p < 0.01$.

S13 Table. Robustness of Results on Distance with Continuous AQI Measures: 1.5 Phones per User

	Full Sample				Weather Subsample		
	(1)	(2)	(3)	(4)	(5)	(6)	(7)
$AQI_{j,y,t}$	-7.906***	-9.146***	-7.424***		-7.592***	-8.177***	-7.166***
	(0.649)	(0.720)	(0.510)		(0.836)	(0.883)	(0.701)
$t \times AQI_{j,y,t}$		0.537***				0.389***	
		(0.101)				(0.145)	
$t^2 \times AQI_{j,y,t}$		-0.028***				-0.020**	
		(0.007)				(0.010)	
$PM_{2.5j,y,t}$			-5.944***				-5.021***
			(0.701)				(0.928)
$AQI_{j,y,t-1}$ (lag)				-7.466***			
				(0.605)			
σ	6348.715***	6348.553***	6349.034***	6348.797***	6283.165***	6283.073***	6284.768***
	(56.709)	(56.715)	(56.860)	(56.708)	(95.434)	(95.444)	(95.622)
Weather Controls	No	No	No	No	Yes	Yes	Yes
N	386,520,317	386,520,317	385,274,973	386,520,317	144,942,148	144,942,148	144,527,552

Notes: This table presents the estimates of Eq (6), with the dependent variable being the distance from home. An observation is an individual in city j on day y in hour t. Each column is a regression, and every regression includes fixed effects $\gamma_{j,y}^{(D)}$ (average willingness to travel in city j on day y) and $\delta_t^{(D)}$ (average willingness to travel at time t). Columns (5)–(7) also include two sets of dummy variables on weather—one set captures the weather categories in Panel A of S4 Table; the other captures the wind speed categories in Panel B of S4 Table. Heteroskedasticity robust standard errors are in parentheses. * $p < 0.10$, ** $p < 0.05$, *** $p < 0.01$.

S14 Table. Robustness of Results on Distance with Continuous AQI Measures: 2 Phones per User

	Full Sample				Weather Subsample		
	(1)	(2)	(3)	(4)	(5)	(6)	(7)
$AQI_{j,y,t}$	-7.905***	-9.144***	-7.422***		-7.592***	-8.178***	-7.166***
	(0.649)	(0.720)	(0.510)		(0.836)	(0.883)	(0.702)
$t \times AQI_{j,y,t}$		0.537***				0.390***	
		(0.101)				(0.145)	
$t^2 \times AQI_{j,y,t}$		-0.028***				-0.020**	
		(0.007)				(0.010)	
$PM_{2.5j,y,t}$			-5.945***				-5.021***
			(0.701)				(0.929)
$AQI_{j,y,t-1}$ (lag)				-7.465***			
				(0.605)			
σ	6348.723***	6348.561***	6349.042***	6348.805***	6283.216***	6283.125***	6284.813***
	(56.706)	(56.712)	(56.857)	(56.705)	(95.426)	(95.436)	(95.614)
Weather Controls	No	No	No	No	Yes	Yes	Yes
N	289,890,212	289,890,212	288,956,212	289,890,212	108,706,659	108,706,659	108,395,709

Notes: This table presents the estimates of Eq (6), with the dependent variable being the distance from home. An observation is an individual in city j on day y in hour t. Each column is a regression, and every regression includes fixed effects $\gamma_{j,y}^{(D)}$ (average willingness to travel in city j on day y) and $\delta_t^{(D)}$ (average willingness to travel at time t). Columns (5)–(7) also include two sets of dummy variables on weather—one set captures the weather categories in Panel A of S4 Table; the other captures the wind speed categories in Panel B of S4 Table. Heteroskedasticity robust standard errors are in parentheses. * $p < 0.10$, ** $p < 0.05$, *** $p < 0.01$.
